# SNPannotator: automated functional annotation of genetic variants and linked proxies

**DOI:** 10.1093/bioinformatics/btag603

**Published:** 2026-08-24

**Authors:** Alireza Ani, Ilja M Nolte, Zoha Kamali, Harold Snieder, Ahmad Vaez

**Affiliations:** Department of Bioinformatics, Isfahan University of Medical Sciences, Isfahan, 8174673461, Iran; Department of Epidemiology, University of Groningen, University Medical Center Groningen, Groningen, 9700 RB, The Netherlands; Department of Epidemiology, University of Groningen, University Medical Center Groningen, Groningen, 9700 RB, The Netherlands; Department of Bioinformatics, Isfahan University of Medical Sciences, Isfahan, 8174673461, Iran; Department of Epidemiology, University of Groningen, University Medical Center Groningen, Groningen, 9700 RB, The Netherlands; Department of Epidemiology, University of Groningen, University Medical Center Groningen, Groningen, 9700 RB, The Netherlands; Department of Bioinformatics, Isfahan University of Medical Sciences, Isfahan, 8174673461, Iran; Department of Epidemiology, University of Groningen, University Medical Center Groningen, Groningen, 9700 RB, The Netherlands

## Abstract

**Summary:**

Genome-wide association studies (GWASs) have identified thousands of genetic variants associated with complex traits and diseases. However, explaining the mechanisms underlying phenotypic variation remains challenging. Here, we introduce *SNPannotator*, an automated post-GWAS analysis software package designed to streamline the interpretation of GWAS findings. Our pipeline implements a multi-step process that identifies proxy variants in high linkage disequilibrium (LD) with associated lead variants, then queries comprehensive resources (including Ensembl, the GTEx Portal, the eQTL Catalog, and STRING DB) for genomic position, deleteriousness, regulatory annotations, clinical significance, trait associations, expression (eQTLs) and splicing quantitative trait loci (sQTLs), and functional enrichment analyses and compiles the results into user-friendly reports. This package is implemented in the R programming language and includes auxiliary functions for variant lookup and LD exploration. *SNPannotator* provides a practical framework for efficiently deriving biologically meaningful insights from GWAS data and for assisting researchers in prioritizing candidate variants for functional validation.

**Availability and implementation:**

The *SNPannotator* package is available from the Comprehensive R Archive Network (CRAN) at https://cran.r-project.org/web/packages/SNPannotator. The development version and tutorial is available on GitHub (https://github.com/omicslaboratory/SNPannotator). The online version of the package is available at https://omicslab.org/snpannotator.

## 1 Introduction

Despite the success of genome-wide association studies (GWAS) in identifying genetic variants associated with heritable phenotypes, translating these findings into biological meaning remains a major challenge (Uffelmann *et al.* 2021, Bruner and Grant 2024). This difficulty is due to multiple factors, such as ancestral diversity, polygenicity, small effect sizes, pleiotropy, and the predominance of non-coding and regulatory variants among the identified variants (Schipper and Posthuma 2022). GWAS-identified variants are also often in high linkage disequilibrium (LD) with numerous other variants, which precludes direct biological or causal interpretation. Therefore, comprehensive analysis aimed at functionally annotating lead variants of the associated loci is essential (Pierce *et al.* 2020).

Multiple tools and resources have been developed for the functional annotation of genetic variants and can be used for post‑GWAS interpretation. These have substantially enhanced our understanding of the genetic architecture of complex traits and disorders. However, most depend on developer‑provided reference databases requiring large periodic updates, generate voluminous outputs, and can yield divergent difficult to interpret results (Yang and Wang 2015, Tuteja *et al.* 2022, Bombarda *et al.* 2025, Pilalis *et al.* 2025). Moreover, these tools may be limited by outdated data, as updating their internal databases with the latest published biological resources is often technically complex and time-consuming. ANNOVAR (Wang *et al.* 2010), MAGMA (de Leeuw *et al.* 2015), and DEPICT (Pers *et al.* 2015) are among the most widely used standalone tools. FUMA, on the other hand, is a web-based platform that integrates tools like ANNOVAR and MAGMA within a unified pipeline to prioritize candidate genes and biological pathways from GWAS findings (Watanabe *et al.* 2017). For this pipeline, GWAS summary statistics should be uploaded on the FUMA server, where processing times may vary depending on job queues, and repeating analyses under different parameter settings can therefore be challenging.

Another approach, described as *in-silico* sequencing (Vaez *et al.* 2015), suggests a systematic workflow to investigate the functional impact of genetic variants. It has been previously used to gain biologically relevant insights from several GWAS studies including those on serum C-reactive protein level (Vaez *et al.* 2015), human reproductive behavior (Barban *et al.* 2016), heart rate variability (Nolte *et al.* 2017), glaucoma (Asefa *et al.* 2022), arterial calcification (De Haan *et al.* 2022), and blood-pressure traits (Keaton *et al.* 2024). However, this pipeline requires multiple manual steps and may not be easily accessible to all researchers. Users must have access to various large reference data and be familiar with several tools including VCFtools (Danecek *et al.* 2011), PLINK (Purcell *et al.* 2007), ANNOVAR, and the GWAS Catalog (Sollis *et al.* 2023).

Here we introduce *SNPannotator*, an automated and user-friendly software package based upon the previously mentioned *in-silico* sequencing pipeline. This tool is designed to simplify post-GWAS functional annotation and to facilitate the extraction of biologically meaningful insights from GWAS hits.

## 2 Methods

*SNPannotator* is developed in R and is available on both the Comprehensive R Archive Network (CRAN) and GitHub. It is designed to be user-friendly, and all options and parameters are controlled through one configuration file. The pipeline utilizes online Application Programming Interfaces (API) to ensure access to the most current data. When supported by the server and to speed up data retrieval, batch requests are used to submit multiple ids in a single request.

For the *in-silico* sequencing approach, the user provides a list of genetic variants (e.g. GWAS top rsIDs) for functional annotation. First, variants in high LD with the input variants are identified using the Ensembl server (Dyer *et al.* 2025). This step is based on the genomic build (GRCh37 or GRCh38), population (e.g. European), window size (in kilobases), and *r*^2^ threshold, all of which are set by the user. The resulting list of primary and proxy variants is then queried across various resources, including the Ensembl server, STRING DB (Szklarczyk *et al.* 2023), the GTEx Portal (Lonsdale *et al.* 2013), and the eQTL Catalog (Kerimov *et al.* 2023) to retrieve relevant data. The gathered data are merged and analysed and the final annotated results are saved for further investigation.

**Figure 1:**
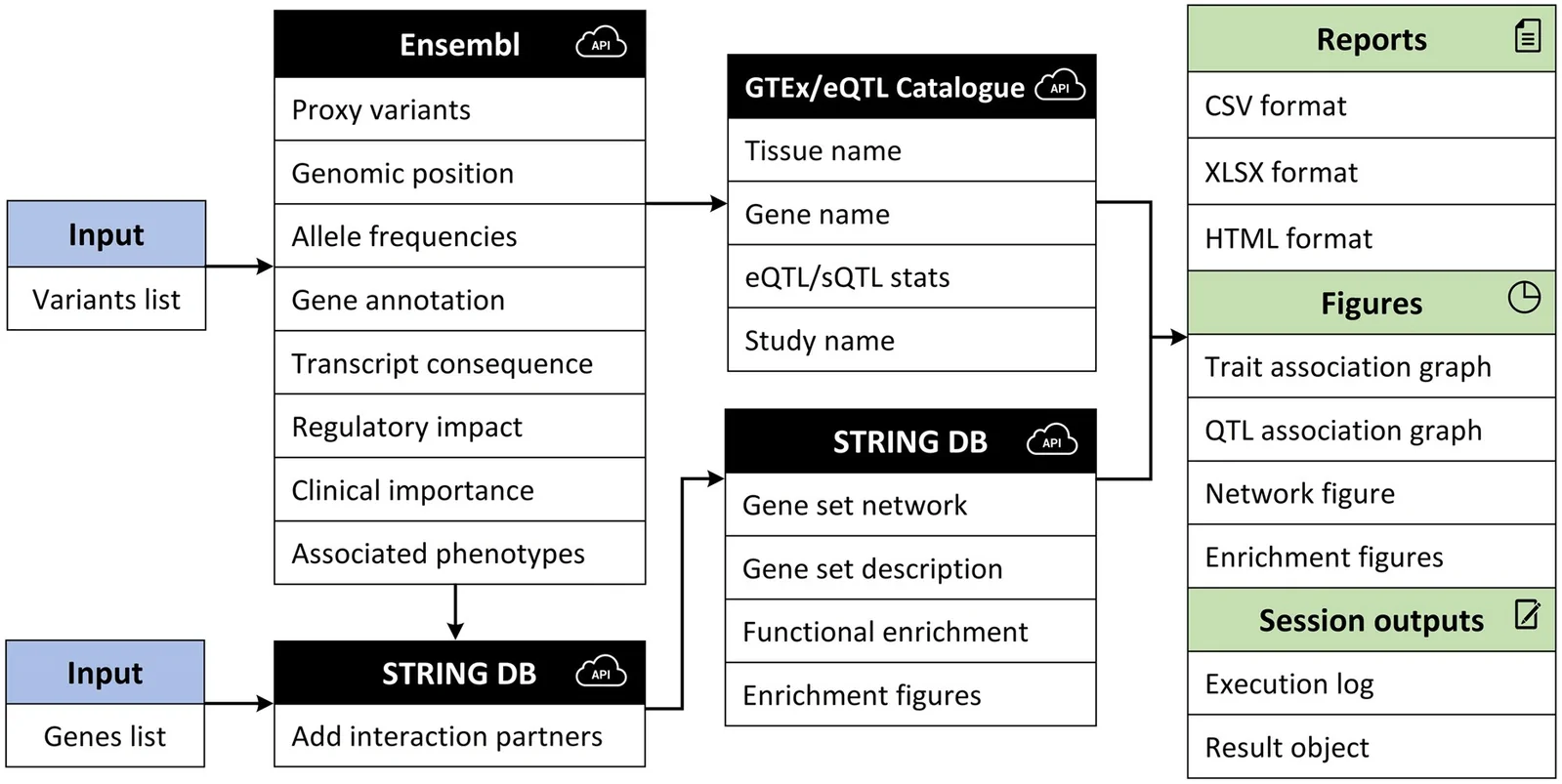
Overview of the *SNPannotator* workflow, analyses, and reports. The *in-silico* sequencing pipeline requires two input files, a list of variant rsIDs and a configuration file specifying runtime parameters. Primary and proxy variants are then queried across multiple resources. The collected data are merged, analysed, and saved as annotated results for further interpretation. Additionally, functional enrichment analysis can be performed on user-selected genes using the STRING DB server.

As an additional feature, the user can provide a list of gene names for enrichment analysis. This list is used to query STRING DB for interaction partners according to user-defined parameters, such as significance threshold and maximum number of partners to be added. The resulting extended list is subsequently subjected to functional enrichment analysis in STRING DB to identify significantly overrepresented biological processes, pathways, molecular functions, etc. Figure 1 presents an overview of the provided functions and package structure.

## 3 Application

A detailed tutorial is available in the Supplementary Material, and the *demo_annotation()* function is prepared for quickly testing one single nucleotide polymorphism (SNP).

Step-by-step instructions for running the package can be found in the tutorial, the package vignette, and the GitHub README file. In brief, for the *in-silico* sequencing pipeline, the user should prepare two files: one containing variant rsIDs, and a configuration file specifying runtime parameters such as the variants file name, the output directory for saving the results, genome build, proxy search parameters, etc. The configuration file is then passed to the *run_annotation()* function to launch the pipeline. The following sections describe the data collected at each stage.

### 3.1 General variant information

Each variant is searched in the Ensembl database using an API call. Information such as the variant’s genomic location, alleles, allele frequency in the selected population, variant type (SNP, indel), and the associated gene is retrieved. Additionally, the deleteriousness, clinical importance, and regulatory features associated with the variant are identified. *SNPannotator* also extracts variants in high LD with the primary variants, based on user-defined *r*² threshold, window size, and population of interest. For these so-called *proxy* variants the same data are collected.

### 3.2 Associated phenotypes

Identifying previously reported variant-phenotype associations provides the biological context for their interpretation. Ensembl offers a comprehensive resource for disease and trait associations by gathering data from multiple sources including ClinVar (Landrum *et al.* 2020) and the GWAS Catalog (Sollis *et al.* 2023). In our pipeline, the primary variants and their proxies are queried against Ensembl using API calls to retrieve known phenotype associations.

Given the many-to-many relationships between traits and variants, the tabular data are transformed into a graph structure using the igraph package (Csardi and Tamas 2006), in which variants and phenotypes are represented as interconnected nodes. Network clustering is then performed using the Louvain algorithm from the same package, which partitions the graph into modules based on connectivity patterns. The final clustered network, with clusters displayed in distinct colors, is exported as a figure and a structured Excel sheet to facilitate further exploration. A sample demonstration is available from the Supplementary Material.

### 3.3 Quantitative trait loci lookups

Primary variants and their proxies are automatically searched in the GTEx Portal (Lonsdale *et al.* 2013) or the eQTL Catalogue (Kerimov *et al.* 2023) using API calls to investigate their potential regulatory effects on gene expression and splicing across multiple tissues and cell types (i.e. expression and splicing quantitative trait loci [eQTLs and sQTLs, respectively]). For each variant key information including biological category or functional classification of the gene, the tissue studied, effect size, standard error, and *P*-value for each association is retrieved to show the magnitude and statistical significance of its regulatory impact. A graph is then constructed from gene-variant association data using the igraph package, in which genes and variants are represented as nodes, and their associations as edges. Similar to the trait association graph, the Louvain method is used to cluster genes and structure the results for reporting.

### 3.4 Functional enrichment analysis

A gene set is automatically created from the genes associated with the primary variants and their proxies. This list is then expanded by including interaction partners through STRING DB using an API call. Threshold of significance to include an interaction and the maximum number of interaction partners can be controlled by the user by parameter settings in the configuration file. This final gene set is then used for additional analyses, including gene set description, functional enrichment analysis and visualizations through STRING DB. A link to the STRING DB website is also included so that users can continue the analysis or make new plots online.

### 3.5 Output files

The software automatically generates well-defined reports in structured HTML and Excel format. Graphical images and text files are also stored. The HTML report is the most comprehensive, and contains variant information, trait associations, regional plots, network visualization, and enrichment figures. The Excel file includes multiple spreadsheets, each summarizing different result such as the annotation report, e- and sQTL findings, trait association clusters, functional enrichment results, and gene descriptions. A log file records detailed steps of the execution and all encountered warnings or errors. An R object file stores all data including configuration settings, input list and original information retrieved from external servers.

### 3.6 Other functionalities

*SNPannotator* offers other practical features for genetic variant analysis using data from the Ensembl database and GTEx Portal, beyond the *in-silico* sequencing pipeline and enrichment analysis. These include identification of variants in high LD with specified loci, computation of LD between multiple variants, finding variants within a specified genomic region, finding synonyms for a given rsID, and determination of the genomic position attributed to an rsID. Both GRCh37 and 38 genome builds are supported. These functionalities offer a more rapid alternative compared to accessing the corresponding websites and users can efficiently retrieve and save outputs directly from within the R environment.

## 4 Results

*SNPannotator* has already been successfully applied in the downstream analysis of several GWAS studies, including published research (Lu *et al.* 2024, Rosenberg *et al.* 2024), and manuscripts under peer review. As a case presentation, 22 SNPs associated with depression (Amare *et al.* 2020) were analysed. In the original report, these variants were annotated using an earlier version of the pipeline, which required multiple manual steps, and were used to gain insights into the interpretation and understanding of their GWAS results. Here, the same variants were reanalysed using the automated pipeline and the output was compared with FUMA. The results are provided as a supplementary document. Sample HTML and Excel file are also included in the Supplementary Material as examples of package output.

## 5 Discussion

*SNPannotator* offers key advantages over the existing resources. First, by accessing data sources through APIs, the pipeline ensures that analyses are based on the most current information available from data providers. This contrasts with other software, where data updates depend on periodic releases from developers, which may lag behind the latest data.

Second, this software significantly minimizes local storage requirements by retrieving variant-related data directly from online database providers in real time. This approach eliminates the need to download and maintain large reference datasets locally.

Third, the entire analysis is performed within the R programming environment, eliminating the need for users to install and learn multiple external tools. Users are only required to configure basic parameters and provide minimal input, making the software accessible to researchers with varying levels of computational expertise.

Fourth, the pipeline enables direct identification of variants in high LD with variants (e.g. GWAS top hits) across different ethnicities using the Ensembl server. This integration removes the need for additional databases or software for LD analysis.

Finally, the workflow is optimized for scenarios where only the GWAS top hit variants are available, and does not require GWAS summary statistics, further simplifying the input requirements.

A limitation of this package is its reliance on continuous API calls, which necessitates a stable internet connection throughout execution. Additionally, it depends on the availability of external service providers. However, considering the reputation and consistent performance of the used API servers, downtime has been rare and their service has remained reliably accessible.

In conclusion, *SNPannotator* is a new, robust, and user-friendly R package to facilitate post-GWAS analysis through automated functional variant annotation. Its applicability extends to other omics frameworks, such as epigenome-wide or transcriptome-wide association studies, when a list of significantly associated genes is provided as input. Our publicly accessible web portal (https://omicslab.org/snpannotator) provides online search capabilities and an overview of the package’s functionalities. By integrating multiple biological resources into a streamlined workflow, *SNPannotator* supports researchers seeking to translate genetic associations into biological knowledge, resulting into improved understanding of complex traits and diseases.

## Supplementary Material

btag603_Supplementary_Data
