# Supplementary material for: SNPannotator: automated functional annotation of genetic variants and linked proxies
Source: Bioinformatics. 2026 Aug 24;42(9):btag603. doi: 10.1093/bioinformatics/btag603 (PMC13533565; doi:10.1093/bioinformatics/btag603)
Supplement: Supplementary file 1 [file btag603_supplementary_data.zip › SNPannotator_manual_R2.docx]

**Introduction to “SNPannotator” package**

Version: 1.4.8

Date: 2026-06-27

Developed by: Alireza Ani, Ahmad Vaez

**Table of contents**

[**Introduction**	3](#_Toc233208171)

[**How it works**	3](#_Toc233208172)

[**Connecting to external databases**	3](#_Toc233208173)

[**Using a local server or offline data**	3](#_Toc233208174)

[**Working with large variant lists**	4](#_Toc233208175)

[**How to install the package**	4](#_Toc233208176)

[**Package dependency list**	6](#_Toc233208177)

[Pandoc module for generating HTML report	6](#_Toc233208178)

[Verifying Pandoc availability in R	7](#_Toc233208179)

[**Input Preparation**	8](#_Toc233208180)

[**Setting parameters in configuration file**	8](#_Toc233208181)

[**Best practices for setting the LD r^2^ threshold**	9](#_Toc233208182)

[**Variant-to-Gene Mapping**	10](#_Toc233208183)

[**Step by step guide to running the package**	13](#_Toc233208184)

[**Annotation steps of the top GWAS variants**	13](#_Toc233208185)

[Step 1: Identifying variants in high linkage disequilibrium (LD)	14](#_Toc233208186)

[Step 2: Obtaining variant information	14](#_Toc233208187)

[Step 3: QTL Analysis	15](#_Toc233208188)

[Step 4: Functional Enrichment Analysis	16](#_Toc233208189)

[**Changing API endpoints**	19](#_Toc233208190)

[**Output files**	21](#_Toc233208191)

[**Annotating Large Numbers of Variants in Multiple Runs**	26](#_Toc233208192)

[**Package functions**	27](#_Toc233208193)

[demo_annotation()	27](#_Toc233208194)

[EnsemblDatabases()	28](#_Toc233208195)

[EnsemblReleases()	29](#_Toc233208196)

[findGenomicPos()	30](#_Toc233208197)

[findPairwiseLD()	31](#_Toc233208198)

[findProxy()	32](#_Toc233208199)

[findRSID()	33](#_Toc233208200)

[findSynonyms()	34](#_Toc233208201)

[getConfigFile()	35](#_Toc233208202)

[run_annotation()	36](#_Toc233208203)

[merge_annotations()	37](#_Toc233208204)

[run_stringdb_annotation()	38](#_Toc233208205)

# **Introduction**

SNPannotator is a bioinformatics tool designed to annotate selected genetic variants, such as top hits from genome-wide association studies (GWAS). While GWAS identifies statistical associations between variants and phenotypes, it does not reveal the biological mechanisms involved. This software facilitates post-GWAS and general variant annotation by assessing the functional impact of variants on human biology. This manual provides a step-by-step guide to using the SNPannotator package, helping researchers interpret the genetic architecture of complex traits and diseases beyond GWAS findings.

# **How it works**

SNPannotator automatically retrieves up-to-date information from multiple well-established biological databases. This method ensured that every analysis is based on the most current findings available without requiring the users to download and maintain large local datasets. So, the users don’t need to worry about storage space or whether the data is outdated. If available, users can also choose which version of the data to use which gives full control over reproducibility. Beyond data retrieval, SNPannotator automatically generates well-structured reports that present results clearly and consistently. These reports are designed to make simpler interpretation of the results.

# **Connecting to external databases**

SNPannotator retrieves data from four major biological databases: Ensembl, GTEx portal, EBI, and STRING-DB. These are well-established, actively maintained resources and are widely used by the research community. Before starting the job, it automatically checks whether the required databases are reachable. If a server is temporarily unavailable, the process stops with a message. In practice, downtime on these servers is rare and brief.

# **Using a local server or offline data**

If you have access to a local copy of the data (e.g. local Ensembl server), you can specify the corresponding API URL in the configuration file. In this case the local or institutional server is used instead of the default external server over the internet, without any changes required to the rest of your analysis.

# **Working with large variant lists**

Post-GWAS analysis is typically performed on a limited number of independent variants, and in most cases your entire list can be processed in a single run without any issues. However, if you have a large number of variants, or if you are working in an environment where the internet connection may be unstable, we recommend splitting your input list into smaller batches of 10 to 20 variants each and running the package separately for each batch. So, if the connection is interrupted, only the affected batch needs to be repeated rather than starting the whole analysis. Once all batches are complete, the built-in merge function can combine all individual outputs into a single, unified result file. See "running a batched analysis" section for details on how to use this feature.

# **How to install the package**

The easiest way to install the SNPannotator package is to get it from the Comprehensive R Archive Network (CRAN). Required dependencies will be downloaded automatically:

install.packages('SNPannotator')

You can also install the latest version from GitHub. In that case, ensure that all dependencies are installed. Use the function below to check for and install any missing dependencies. Also, please ensure the ‘devtools’ package is installed and loaded beforehand:

```
# Verify that all required package dependencies are installed.
# Any packages not found are automatically installed from CRAN.
install_missing_packages <- function() {
  pkgs <- c("data.table","httr","jsonlite","xml2","openxlsx","progress",
            "ggplot2","kableExtra","methods","rmarkdown","ini","igraph",
            "ggraph","logger","png","readr")

  # Identify missing packages
  missing <- pkgs[!sapply(pkgs, requireNamespace, quietly = TRUE)]

  if (length(missing) > 0) {
    message("Installing missing packages: ",
paste(missing,
collapse = ", "))
    install.packages(missing)
  } else {
    message("All required packages are already installed.")
  }
}

# run the function
install_missing_packages()

# Install devtools if not already installed
install.packages("devtools")
# Load devtools package
library(devtools)
# Install the package from GitHub
install_github("omicslaboratory/SNPannotator")
```

Please note that the recommended installation method is via CRAN using install.packages("SNPannotator"), which automatically resolves and installs all required dependencies. Users installing the package through GitHub via devtools::install_github() will similarly benefit from automatic dependency installation. The install_missing_packages() function is provided solely as a convenience utility for users who install the package through alternative methods, such as from a local .tar.gz source file, where automatic dependency resolution may not occur.

**Package dependency list**
Following packages and modules are used in SNPannotator package.

| **Package name** | **Required** | **Description** |
| --- | --- | --- |
| ini | ✓ | Loads the configuration file |
| data.table | ✓ | Fast data manipulation and analysis |
| ggplot2 | ✓ | Powerful and flexible data visualization |
| rmarkdown | ✓ | Dynamic document generation and reporting |
| kableExtra | ✓ | Enhanced table creation and styling |
| openxlsx | ✓ | Excel file reading and writing |
| httr | ✓ | Web API requests and handling |
| jsonlite | ✓ | JSON data parsing and generation |
| xml2 | ✓ | XML parsing and manipulation |
| progress | ✓ | Progress bars for long-running operations |
| methods | ✓ | Object-oriented programming in R |
| igraph | ✓ | Network analysis and visualization |
| ggraph | ✓ | Graph and network visualization |
| logger | ✓ | Logging framework for R |
| png | ✓ | Read and write PNG images |
| readr | ✓ | Fast and friendly data import |
| pandoc | 🗶 | See below |

Table 1. Package dependency list

### Pandoc module for generating HTML report

[Pandoc](https://pandoc.org/) is a free and open-source document converter that is used for creating an easy-to-view HTML report. It is required for creating reports in HTML format; however, if it is unavailable, reports will be generated in text and Excel formats only.

**Note**: Pandoc is automatically installed with RStudio in windows operating systems. However, in some cases manual configuration may still be required depending on the operating system and RStudio version.

**Installation:** Please follow the [instructions](https://pandoc.org/installing.html) on how to install Pandoc on your system. You can also use the 'installr' package on **Windows** operating systems. First, install the 'installr' package and then run the following command.

install.packages('installr')

installr::install.pandoc()

On most Linux distributions, Pandoc can be installed via the system package manager. Note that root privileges are required:

sudo apt-get install pandoc

On macOS, Pandoc can be installed using Homebrew:

brew install pandoc

### Verifying Pandoc availability in R

After installation, it is important to confirm that Pandoc is correctly detected by R and is accessible within your current R session. To check this, run the following commands in the R console:

```
# Check whether Pandoc is available to R
rmarkdown::find_pandoc()
# Check the detected Pandoc version
rmarkdown::pandoc_version()
```

If you are sure of installing Pandoc but rmarkdown::find_pandoc() returns NULL or an empty path, this means R cannot locate your Pandoc installation. In this case, you can manually specify the path to the Pandoc executable using:

Sys.setenv(RSTUDIO_PANDOC = "/path/to/your/pandoc")

Replace “/path/to/your/pandoc” with the actual directory containing the Pandoc executable on your system. On Windows, this path is typically “C:/Program Files/Pandoc”. On Linux or macOS, the path can be found by running “which pandoc” in the terminal.

**Note:** If generateHTML = TRUE was set in the configuration file but no HTML report was produced, please check the log file generated during the run. Any Pandoc-related errors or warnings will be explicitly recorded.

# **Input Preparation**

SNPannotator requires two input files:

1. **List of genetic variants:** A text file containing the list of variants, such as top independent GWAS variants. Each line of this file corresponds to a single rsID, which represents a genetic locus associated with the studied trait.

If the rsID is not known, it can be retrieved from genomic position using the *findRSID()* function in this package. Please refer to the function parameters for more details.

2. **Configuration File:** The package includes a template configuration file for specifying run-time parameters. Before running an analysis, users should copy this file to a directory of their choice and customize it as required. The following command copies the embedded template file and saves it as *inSilicoSeqConfig.ini* in the specified directory:

```
# first, load the library
library(SNPannotator)
# 1- save the configuration file to a specific directory
getConfigFile('/home/user/project1/postGWAS')
# 2- OR save to the current working directory
getConfigFile('.')
```

# **Setting parameters in configuration file**

SNPannotator uses a configuration (.ini) file to configure the pipeline specifications. As mentioned above, a sample file is included in the package, which should be edited and used for variant annotation.

This file has three components:

1. **Sections:** Keys are grouped into named sections. The section name appears on a line by itself, in square brackets ([ and ]).
2. **Key or property:** these are the basic parameters for configuring the pipeline. Every key has a name and a value, delimited by an equals sign (=).
3. **Comments:** Semicolons (**;**) or hash (**#**) at the beginning of the line indicate a comment. Comment lines are just for clarifying the items’ purpose and will be ignored when running the file.

**Important notes:**

- Key-names and section-names should **NOT** be edited or renamed. Otherwise, the algorithm will not work properly.
- Lines that start with ‘**#**’ character are comments about a parameter. **DO** **NOT** un-comment the information line by deleting this character.
- Properties that start with a ‘**;**’ character are treated as commented-out parameters. In this case, they are ignored and the default value for that parameter is used instead. To enable the parameter, remove the semicolon and assign a value after ‘=’ sign.

**For example:** ;imagetype=image uses the default and can be changed to imagetype=highres_image to set the value explicitly.

- Avoid duplicated keys.
- Spaces before or after the '=' sign are permitted and do not affect file parsing.

# **Best practices for setting the LD r^2^ threshold**

Population selection for LD calculation must match the GWAS cohort ancestry, as LD patterns vary substantially across populations. The r2 threshold parameter controls the minimum level of linkage disequilibrium required for a variant to be returned as a proxy for the target SNP. Selecting an appropriate threshold requires balancing sensitivity against interpretability. Setting a very stringent threshold (e.g., r^2^≥0.9) will return only the most highly correlated proxies and may miss variants of biological relevance. Conversely, setting a very low threshold (e.g., r^2^≤0.5) will substantially increase both the number of returned variants and the overall run time, and may introduce weakly correlated variants that add noise and complicate downstream interpretation. A threshold of r^2^≥0.6 represents a pragmatic lower bound and is considered a reasonable starting point. The recommended approach is to set the threshold to the lowest value you consider biologically justifiable for your specific analysis in order to capture all potentially relevant proxies in a single run, and then apply post-hoc filtering on the r^2^ column in the output Excel file to refine results based on specific LD requirements. This avoids the need to repeat the analysis multiple times and ensures that no relevant information is discarded prematurely during the annotation step. Users should be aware, however, that lower thresholds will significantly increase run time and the volume of returned results, which may make interpretation more challenging.

# **Variant-to-Gene Mapping**

SNPannotator maps each input variant to one or more genes using Ensembl. The mapping strategy depends on the genomic context of the variant. For variants that fall within a gene body, all overlapping Ensembl genes are retrieved and reported. A single variant may therefore be associated with more than one gene when it overlaps multiple genes or transcripts, and all such associations are retained in the output table. For intergenic variants, the nearest gene is returned by default. The biotype of the nearest gene is specified via the nearestGene_type parameter in the configuration file. Supported options include:

protein_coding, IG_pseudogene, scRNA, translated_processed_pseudogene, IG_J_pseudogene, TR_J_pseudogene, vault_RNA, TR_D_gene, sRNA, TR_C_gene, ribozyme, IG_C_pseudogene, IG_C_gene, IG_J_gene, artifact, TR_V_pseudogene, IG_D_gene, rRNA, scaRNA, TR_J_gene, unitary_pseudogene, TR_V_gene, IG_V_gene, transcribed_unitary_pseudogene, IG_V_pseudogene, rRNA_pseudogene, transcribed_processed_pseudogene, transcribed_unprocessed_pseudogene, snoRNA, TEC, miRNA, snRNA, misc_RNA, unprocessed_pseudogene, processed_pseudogene, lncRNA

For example, setting nearestGene_type = "protein_coding" ensures that the nearest protein-coding gene is assigned to each intergenic variant.

**Note:** Nearest-gene assignment for intergenic variants is a proximity-based heuristic and does not imply a functional relationship between the variant and the assigned gene. When a substantial proportion of input variants are intergenic, downstream gene-set enrichment results should be interpreted with caution, as the gene list is partly determined by genomic proximity rather than direct annotation.

Table 2. Configuration file sections and properties

| **Section** | **parameter** | **default value** | **Description** |
| --- | --- | --- | --- |
| general | outputFolder |  | Where to save reports, active directory will be used by default |
|  | projectName |  | The name that will be used for saving output files |
|  | inputFile |  | The list of variants to be looked up. Each rsID should be provided on a separate line in the input file. |
|  | db^*^ | 1000GENOMES:phase_3:EUR | The population that should be used for LD calculation based on Ensembl data. |
|  | window_size | 500 | Number of base pairs around the variant for checking LD scores (accepted range: 100–500 kb, inclusive). |
|  | r2 | 0.8 | The LD threshold for selecting variants around the target SNP. |
|  | LDlist | FALSE | Whether variants in high LD should be found and added to the output |
|  | nearestGene_type | all | Type of nearest gene to return.  "all" will return whichever gene was nearest. |
|  | generateHTML | TRUE | Whether to save an html report. |
| API | ENSEMBL_API_ENDPOINT | https://rest.ensembl.org | Access genomic data from Ensembl |
|  | EBI_API_ENDPOINT | https://www.ebi.ac.uk/eqtl/api/v3 | Access eQTL data from EBI |
|  | GTEX_API_ENDPOINT | https://gtexportal.org/api/v2 | Access QTL data from GTEx Portal |
|  | STRING_API_ENDPOINT | https://string-db.org/api | Access data from STRING-DB |
| STRING_DB | functional_enrichment | TRUE | Whether to get functional enrichment data. |
|  | network_image | TRUE | Retrieves the network image with input genes. |
|  | webLink | TRUE | Save the weblink in output files. |
|  | description | TRUE | Getting the description of the genes |
|  | imagetype | image | Network PNG image (image/highres_image/svg) |
| QTL | eQTL | TRUE | Search eQTL information |
|  | sQTL | TRUE | Search sQTL information |
|  | dataset | gtex_v10 | The database to be used for eQTL/sQTL mapping in GTEx Portal. Either gtex_v8 or gtex_v10 is accepted |
|  | pvalue_threshold | 5E-5 | P value threshold for filtering the eQTL results |
| graphs | generate_graph | TRUE | Present output of eQTL and GWAScatalog as graphs |
|  | layout | layout_nicely | Graph layout from igraph package, layout_in_circle , layout_nicely, etc. |

^*^ The most commonly used database options for the 1KG project are listed below. Use the EnsemblDatabases() function to view all available options.

1000GENOMES:phase_3:ALL

1000GENOMES:phase_3:EUR

1000GENOMES:phase_3:AFR

1000GENOMES:phase_3:AMR

1000GENOMES:phase_3:YRI

# **Step by step guide to running the package**

1- **Create an empty folder** to save the output result files. Ensure that current user and R environment has the necessary read/write permissions for this folder. Note that the folder is not created automatically and must exist beforehand.
2- **Prepare the variants input file.** Each line of the file should correspond to a single variant rsID. It is recommended to place this file in the newly created folder for easy access. If the rsID is not known, it can be retrieved from genomic position using the *findRSID()* function in this package. Please refer to the function parameters for more details.
3- **Obtain the configuration file** from the package using the command getConfigFile('/path/to/folder'). It is recommended to copy this file into the newly created folder for easy access.
4- **Modify the parameters** in the configuration file as needed.
5- **Run the annotation pipeline** by running the command run_annotation('/path/to/ inSilicoSeqConfig.ini').

# **Annotation steps of the top GWAS variants**

The steps included in this pipeline are:

- Identifying variants in linkage disequilibrium (LD) with top GWAS hits (optionally)
- Collecting variant information and their regulatory potential
- Finding the previously found associated traits
- Gathering the QTL data
- Conducting enrichment analysis to understand broader biological networks

Please note that if the user selects GRCh38, the GTEx Portal will be queried, which provides both eQTL and sQTL data. However, if GRCh37 is selected, the eQTL Catalogue will be searched, which only contains eQTL data due to data provider limitations. Since the GTEx Portal is faster and offers more comprehensive information, build 38 is highly recommended.

## Step 1: Identifying variants in high linkage disequilibrium (LD)

If the "LDlist" parameter is set to "TRUE" in the configuration file, the package retrieves variants in LD with the top GWAS variants. Data are directly fetched from Ensembl server.

Below options are used for this purpose:

- **server**, This option can be set to query either GRCh37 or GRCh38 via the API; GRCh38 is recommended and is the default.
- **db**, the population data to be used for calculating LD between variants. 1000GENOMES:phase_3:EUR is selected by default. Please refer to footnote in Table 2 for more information and options.
- **window_size**, Number of kilobases (Kb) around the variant used for calculating LD scores. Acceptable values range from 0 to 500, with a default of 500.
- **r2**, LD threshold for selecting variants around the target SNP. Acceptable values range from 0.5 to 1.0, with a default of 0.8.

If the LDlist parameter in the configuration file is set to FALSE, only the variants specified in the input list will be used for analysis. By default LDlist is set to TRUE.

## Step 2: Obtaining variant information

Each variant is searched in the Ensembl database (https://www.ensembl.org). Information including the variant's physical location on the genome, its alleles, allele frequencies in selected population, variant type (e.g., SNP, indel), and the nearest gene are obtained. Additionally, the most severe consequence, deleteriousness, and regulatory features of the genomic region containing the variant are identified.

Previously reported variant-trait associations are also retrieved, including data from multiple sources such as ClinVar, GWAS Catalog, and other databases. The complete list of these sources is available at the [following URL](https://www.ensembl.org/info/genome/variation/species/sources_documentation.html). These data are organized into three tables: one for ClinVar, one for GWAS Catalog, and one for associations from sources other than these two. Only significant associations, defined by a reported p-value < 0.05, are included to ensure the reliability of the reported variant-trait links.

## Step 3: QTL Analysis

Top hit variants and proxies (with r^2^ > 0.8) are searched in the GTEx Portal (<https://gtexportal.org>) or eQTL Catalogue (<https://www.ebi.ac.uk/eqtl>) to investigate their potential regulatory effects on gene expression and splicing events. To control the number of associations returned for each lead SNP, a hard limit of 10,000 associations and a pvalue threshold of 0.05 is used. This filtering is applied to avoid an excessive amount of data that would result from including all possible proxies. The result can help identify genetic variants that influence gene regulation mechanisms, such as transcript isoform abundance and alternative splicing, across multiple tissues. Key information, such as the variant's impact on the gene expression and splicing levels, is retrieved. This includes the effect size, which quantifies the magnitude of the variant's influence, and the associated p-value, which indicates the statistical significance of this effect. These data are collected across various tissues and cell types, providing insights into tissue-specific regulatory roles of the variant on both gene expression and RNA splicing. Network clustering is performed using the Louvain algorithm from the igraph package, which partitions the graph into modules based on connectivity patterns. Edges are unweighted, and no additional filtering is applied before clustering. Because the Louvain algorithm is stochastic, a fixed seed is set in the code prior to execution to ensure reproducibility across runs.

As previously mentioned, if the user selects GRCh38, the GTEx Portal will be queried, which provides both eQTL and sQTL data. However, if GRCh37 is selected, the eQTL Catalogue will be searched, which only provides eQTL data due to data provider limitations. Considering that, the GTEx Portal is faster and offers more comprehensive information, **using build 38 is highly recommended.**

The results are provided as two Excel sheets. The first sheet contains the raw output from the data providers (GTEx Portal or eQTL Catalogue). The second sheet presents summary statistics, including a frequency table for variants, genes, or tissues with their counts in the data, as well as the results of clustering analyses.

## Step 4: Functional Enrichment Analysis

A gene set is created by mapping each variant to its associated gene(s) based on Ensembl annotation data (intergenic variants are mapped to the nearest gene), and this unique set of genes is then analyzed using the STRING-DB application (https://string-db.org). The initial gene list is expanded by including interaction partners identified through STRING-DB. If the number of input genes exceeds 250, no additional genes are added. Otherwise, interaction partners are included based on a threshold of 0.700. This threshold represents a high confidence level, indicating that the interactions included have strong supporting evidence and a high likelihood of being biologically relevant.

To control the number of added partners and balance network complexity, the following limits are applied based on the size of the input gene set:

- If the input gene count is between 100–250, a maximum of only 2 interaction partners per gene is included to prevent too many interactions.
- If the input gene count is between 50–100, a maximum of 5 interaction partners per gene is included.
- If the input gene count is below 50, a maximum of 10 interaction partners per gene is included.

The final gene set is then used for further analysis, which involves the following steps:

1) **STRING-DB Network Visualization**: An image of the STRING-DB network showing interactions in the neighborhood surrounding these genes is generated and saved.
2) **Gene Set Description:** A detailed description of the gene set is obtained.
3) **Functional Enrichment Analysis:** Functional enrichment analysis is performed on the gene set to identify enriched biological categories.
4) **Enrichment Figures:** Enrichment figures are generated for specific categories, including Function, Process, Component, Diseases, and Tissues.
5) **STRING-DB Network Link:** A direct link to the STRING network analysis is generated and reported in the output files, allowing users to visit the STRING website and modify parameters or visualize results with highly customizable settings online. This approach provides users with a standardized starting point using widely accepted defaults, while preserving full flexibility for further customization through STRING's web interface.

All results, including network images and enrichment figures for each category, are saved to facilitate downstream analyses and interpretation.

Users should be aware that the composition of the input gene set and consequently the enrichment results is inherently dependent on upstream analytical choices, including the selected LD r^2^ threshold, the window size, and other parameters. For example, a more permissive r^2^ threshold will include a larger and potentially more diverse set of proxy variants, which may map to additional genes and alter the enrichment profile. Users are therefore advised to interpret enrichment results in the context of their chosen parameters.

**Running a batched analysis**

For users who wish to process large variant lists, or who have an unstable internet connection, SNPannotator supports a batched workflow. Users can split the input variant list into smaller batches of 10 to 20 variants, run the package separately for each batch, and then combine all results into a single unified output using the built-in merge function. The final merged output is equivalent to what would have been obtained if all variants were analyzed together in a single run.

It is very important to note that when running batched analyses, we highly recommend **turning off** the STRING-DB analysis option in the configuration file for each individual batch. This is important because if this analysis is run separately on each batch, the results will reflect only the genes identified in that particular batch, rather than the full picture across all variants. Once all batches are complete, the merge function can be used to combine the outputs in a structured way:

- Main reports from all batches are merged into a single report covering all input variants.
- QTL analysis results are combined across batches, and the summary statistics, including the variants, genes, and tissues with the highest number of associations, are fully recalculated based on the complete merged dataset.
- STRING-DB analysis is then run once on the complete gene set extracted from all batches combined. This ensures that the protein interaction network and enrichment results are based on the full and representative set of genes identified across all variants.

**Steps for running a batched analysis**

1. Prepare your input files. Split your full list of input variants into smaller files, each containing 10 to 20 variants (e.g. “BD_proj_1.txt”, “BD_proj_2.txt”, etc.)

2. Configure each batch. Open the configuration file for each separate run and set the `projectName` field to a clear, numbered name that reflects the batch, for example `BD_proj_1`,`BD_proj_2`, and so on. Set the `inputFile` field to point to the corresponding input file for that batch. Make sure the STRING analysis option is turned off at this stage. this can be done by setting all parameters in the [STRING_DB] section to “FALSE” (e.g. functional_enrichment = FALSE, etc.)

3. Run each batch separately. Execute the run_annotation() function for each batch in turn. Each run will produce its own output set of files that are named according to the `projectName` you assigned.

4. Merge the results. Once all batches have finished successfully, use the ` merge_annotations()` function to combine all individual outputs into a single unified result. The function will merge the main reports and QTL results, recalculate summary statistics across the full dataset, and run STRING analysis on the complete gene set. Please refer to the function documentation for a full description of the required parameters.

# **Changing API endpoints**

By default, the package is set to access the latest API endpoints for each supported data provider including Ensembl, eQTL Catalogue, GTEx Portal, and STRING-DB. This approach ensures that you receive the most up-to-date data and features.

However, users have the option to change the default API URLs via the configuration file. This can be useful if you wish to access data from a previous release, or to connect to a locally hosted database (for example, an internal Ensembl REST server).

Previous version of Ensembl data can be selected using below URLs. More information about the possible endpoints are available from: <https://ensembl.org/Help/ArchiveList>

- v115 ==> https://rest.ensembl.org ### the current release for GRCH38
- v114 ==> https://may2025.rest.archive.ensembl.org
- v113 ==> https://oct2024.rest.archive.ensembl.org
- v112 ==> https://may2024.rest.archive.ensembl.org
- https://grch37.rest.ensembl.org ### the latest release for GRCH37

Previous version of STRING-DB data can be selected using below URLs

- <https://string-db.org/api> ### the current release
- <https://version-12-0.string-db.org> ### Similar to above and the current release
- <https://version-11-0.string-db.org>

These are the latest available API endpoints from EBI and GTExportal. **DO NOT CHANGE** unless you have a local repository with the same version:

- <https://www.ebi.ac.uk/eqtl/api/v3>
- <https://gtexportal.org/api/v2>

# **Output files**

After conducting a comprehensive search across multiple databases for variants and their proxy lists, several output files are generated, including text files (.txt), HTML (.html), Excel spreadsheet (.xlsx), and images. The following list provides an overview of each output file, along with detailed descriptions and information about their contents.

Table 3. Output files and their description

| **File description** | **extension** | **description** |
| --- | --- | --- |
| log file | log | Summary of the annotation process including any warnings or errors. |
| Result file | tsv / xlsx | Annotation report in tabular format. |
| Result file | HTML | Annotation report including graphs and data (generated if pandoc module is available). |
| Not found variants | txt | List of variants that were not found. |
| STRING-DB enrichment report | tsv | Enrichment report in tabular format. |
| eQTL report | tsv | eQTL report in tabular format. |
| eQTL association graph | png | Graph of eQTL associations. |
| sQTL report | tsv | sQTL report in tabular format. |
| sQTL association graph | png | Graph of sQTL associations. |
| Trait association graph | png | Graph of trait associations. |
| Network image | png | Network image from STRING-DB. |
| Enrichment figure (function) | png | Enrichment image from STRING-DB. |
| Enrichment figure (process) | png | Enrichment image from STRING-DB. |
| Enrichment figure (component) | png | Enrichment image from STRING-DB. |
| Enrichment figure (disease) | png | Enrichment image from STRING-DB. |
| Enrichment figure (tissue) | png | Enrichment image from STRING-DB. |
| R object file | rds | Input data and results as R data object. |

1- Structure of the output report file (*_output.tsv):

| **Header** | **Description** |
| --- | --- |
| #gSNP | Input variant number |
| gSNP | rsID of the input variant |
| Linked_SNP | rsID of the proxy variant |
| Chr | Chromosome number |
| Pos | Position of the variant on chromosome |
| Class | Variant type (SNV/indel, …) |
| Ref_Allele | Reference allele |
| Alt_Allele | Alternate allele |
| Allele_frequency | Variant allele frequency in the selected population |
| LD | Calculated r^2^between the input and proxy variant |
| Cytoband | Cytoband of the variant |
| Most_severe_consequence | Most impactful predicted effect of a variant on gene function among all possible consequences across different transcripts. This is determined using a subjective ranking system developed by Ensembl, which orders consequences from most severe to least severe based on their potential impact on gene function. |
| Gene | The gene on which the variant is located. For intergenic variants, it is defined as the nearest gene to the variant's position. |
| GeneId | Gene id in Ensembl (ENSG) |
| geneBiotype | Biological category or functional classification of a gene (protein-coding, pseudogene, …) |
| RegId | Identifier for the specific regulatory feature in Ensembl (ENSR) |
| RegType | The regulatory feature of the genomic region where the variant is located (enhancers, promoters, CTCF-binding sites, …) |
| CADD | The Combined Annotation Dependent Depletion score is a computational metric that predicts the deleteriousness of genetic variants |
| SIFT_prediction | SIFT (Sorting Intolerant From Tolerant) is a tool used to predict the potential impact of amino acid substitutions on protein function (“tolerated”, “deleterious”). |
| Polyphen_prediction | A tool used for defining the potential impact of amino acid substitutions on protein structure and function ("probably damaging," "possibly damaging," "benign," or "unknown”) |
| Phenotype | Associated traits with the variants in GWAS catalog |

2- Structure of the eQTL/sQTL report file (*_eQTL/sQTL.tsv):

| **Header** | **Description** |
| --- | --- |
| #gSNP | Input variant number |
| rsId | rsID of the variant |
| Gene | Gene symbol |
| GeneId | Gene id in Ensembl (ENSG) |
| Tissue | Tissue in which the expression of gene has been studied |
| Beta | The effect size of a genetic variant on gene expression |
| pValue | P-value of the association. |

3- Structure of the enrichment report file (*_enrichment.tsv):

| **Header** | **Description** |
| --- | --- |
| category | Term category (e.g. GO Process, KEGG pathways) |
| term | Enriched term (GO term, domain or pathway) |
| description | Description of the enriched term |
| p_value | Raw p-value |
| fdr | Adjusted p-value (false discovery rate, FDR) |
| number_of_genes | Number of genes in the input list with the term assigned |
| number_of_genes_in_background | Total number of genes in the background proteome with the term assigned |
| inputGenes | Gene names from the input |
| preferredNames | Common protein names (in the same order as the input Genes) |

4- Structure of the output Excel report file (*.xlsx):

This file compiles data from report files into a tabular format across multiple sheets. The inclusion of specific sheets depends on user specifications, so not all listed sheets may be present.

| **No.** | **Sheet name** | **Information** |
| --- | --- | --- |
| 1 | All Variants | Similar to the report file in text format. The LD column is color-coded using a gradient to visually represent the strength of linkage disequilibrium (LD) |
| 2 | ClinVar | Variants associations in ClinVar (if any) |
| 3 | GWASCatalog | Variants associations in GWASCatalog (if any) |
| 4 | Phenotypes | Variants associations other than ClinVar/GWASCatalog (if any) |
| 5 | eQTL | Similar to the eQTL report file |
| 6 | eQTL report | Summary statistics, including a frequency table for variants, genes, or tissues |
| 7 | eQTL | Similar to the sQTL report file |
| 8 | sQTL report | Summary statistics, including a frequency table for variants, genes, or tissues |
| 9 | Description | Gene descriptions |
| 10 | Func. enrichment. | Similar to the enrichment report file |

5- HTML report file (*.html):

This file combines data for all variants into one document for easy viewing and comparison. It includes information about the top variant and its proxies (if requested), such as associated genes, traits, and variant types. The file also contains images like regional plots, network images, and enrichment figures. Users can right-click on the images and select 'Open in new tab' to view them at full resolution or choose 'Save image as...' to download the file.

6- Figures: multiple figures are generated during the pipeline. The creation of specific plots depends on user specifications, so not all listed images may be included.

| **File name** | **Description** |
| --- | --- |
| *_network.png | Network image (STRING-DB) |
| *_enrichment_process.png | Enrichment visualization (Biological Process, STRING-DB) |
| *_enrichment_tissues.png | Enrichment visualization (Tissue Expression, STRING-DB) |
| *_enrichment_component.png | Enrichment visualization (Cellular Component, STRING-DB) |
| *_enrichment_disease.png | Enrichment visualization (Disease-gene Associations, STRING-DB) |
| *_enrichment_function.png | Enrichment visualization (Molecular Function, STRING-DB) |
| *_gwascatalog.png | Graph for variant-trait association |
| *_eQTL.png | Graph for variant-associated gene expression information |
| *_sQTL.png | Graph for variant-associated gene-splicing information |

7- R object file (*.rds):

This file contains all data, including the input variants list, configuration parameters, and output data in R format, structured as a list.

# **Annotating Large Numbers of Variants in Multiple Runs**

It may be inefficient to annotate a large set of genetic variants in a single run. To address this issue, the package allows you to split the annotation process into multiple runs, each covering a manageable subset (typically 10–15 variants per run).

*Step-by-Step Workflow*

1- **Split Input Files**
   Divide your variant list into several smaller input files, each containing only a portion of the variants.
2- **Configure Project Names**
   In your configuration file, set the projectName parameter to a unique, numbered name for each run (for example: project_part_1, project_part_2, etc.).
3- **Run the Annotation Process**
   Run the annotation workflow for each subset, using its corresponding input and project name.
4- **STRING Annotation and Enrichment (Recommended Practice)**
   To avoid redundant or fragmented enrichment analysis, it is recommended to turn off STRING annotation and enrichment for each individual run. You can do this by setting the relevant parameter to FALSE in the configuration file for each “part”.
5- **Merge Results**
   After completing all runs, use the merge_annotations() function to combine the outputs from each run into a single, comprehensive result.
6- **Final STRING Analysis**
   When merging, set STRING=TRUE in the function parameters. This will run the STRING enrichment and gene annotation over the entire merged dataset, providing a unified enrichment analysis.

# **Package functions**

## demo_annotation()

A data table containing the variant information for testing is returned. Report files are also saved in the current working directory.

**Example**

output = demo_annotation()

**Output**

This function provides a quick way to test the package by generating a sample output for one variant. Report files are saved in the current working directory.

## EnsemblDatabases()

List of available population from Ensembl website (1000 Genomes project).

**Function parameters**

- **server** Ensemble API end point. default: https://rest.ensembl.org

**Example**

output = EnsemblDatabases()

**Output**

A data table is returned that includes the name, description and size of the available populations in 1000Genomes project database.

## EnsemblReleases()

Shows the data release available on this REST API server.

**Function parameters**

- **server** Ensemble API end point. default: https://rest.ensembl.org

**Example**

output = EnsemblReleases()

**Output**

Data release is displayed.

## findGenomicPos()

Retrieves variant information from the GTEx Portal using either an rsID or a variant ID formatted as CHR_POS_REF_ALT. If an rsID is provided, the function returns the corresponding genomic positions in both GRCh37 and GRCh38 builds. When searching for an rsID based on genomic position, the position parameter should be specified according to the **GRCh38** reference genome.

**Function parameters**

- **id,** Character string representing the rsID (e.g., `"rs12345"`) or the variant ID (e.g., "7_24898067_A_G")
- **type,** Character string specifying the type of query. Must be either "rsid" or "varid". Default: "rsid".
- **file_path,** path to a file for saving results as Excell spreadsheet.

**Example**

findGenomicPos('rs121')

findGenomicPos('7_24898067_A_G', type = "varid", file_path = "output.xlsx")

**Output**

Returns a data table containing variant information including:

- rsid: variant id in rsID format
- chromosome: chromosome number
- position_b37: genomic position
- position_b38: genomic position
- ref: reference allele
- alt: alternate allele

## findPairwiseLD()

This function computes the linkage disequilibrium (LD) between the variants using data from the Ensembl website.

**Function parameters**

- **rslist,** A vector of rs numbers.
- **file,** Path to the Excel file for saving search results. This is optional, the result will be just available in R environment if file path is not provided.
- **pairwise,** If TRUE, compute pairwise LD between all elements of a list. If FALSE, computes the LD between first and other elements of the list. Default: FALSE
- **build,** Genome build. Either 37 or 38. Default: 37
- **db,** The population database for calculating LD scores**.**
- **window_size,** Number of base pairs around the variant for checking LD scores (max = 500kb).
- **r2,** The minimum LD threshold for selecting variants around the target SNP. Default: 0.8.

**Example**

output = findPairwiseLD(c('rs234', 'rs10244533'))

**Output**

The Excel report file contains two sheets. The first sheet includes the input data and parameters, and the second sheet presents the output data in a tabular format.

## findProxy()

This function can be used to find variants that are in high LD with a list of variants using data from the Ensembl website.

findProxy(rslist,

file=NULL,

build='37',

db="1000GENOMES:phase_3:EUR",

window_size=500, r2=0.8)

**Function parameters**

- **rslist,** A vector of rs numbers.
- **file,** Path to the Excel file for saving search results. This is optional, the result will be just available in R environment if file path is not provided.
- **build,** Genome build. Either 37 or 38. Default: 37
- **db,** The population database for calculating LD scores**.**
- **window_size,** Number of base pairs around the variant for checking LD scores (max = 500kb).
- **r2,** The minimum threshold for LD with the target SNP. Default: 0.8.

**Example**

output = findProxy("rs234")

output = findProxy(c("rs234", "rs678"))

output = findProxy("rs234", file = 'output.xlsx', build = 38, r2 = 0.9)

**Output**

The Excel report file contains two sheets. The first sheet includes the input data and parameters, and the second sheet presents the output data in a tabular format.

## findRSID()

This function retrieves variant information from Ensembl based on the specified genomic position. It takes the chromosome number, start position, and end position as input parameters and searches for variants within this window, using the specified genomic build. If only the start position is provided, the function automatically sets the end position equal to the start position. This is particularly relevant for SNP variants, where the start and end positions are the same. The function returns all variants found within the defined window.

**Function parameters**

- **chromosome,** Numeric, specifying the chromosome number.
- **start_position,** Numeric, specifying the starting base pair position.
- **end_position,** Numeric, specifying the ending base pair position.
- **build,** Numeric, specifying the genomic build, default value is 38.
- **file_path,** Character, path to a file for saving results as Excell spreadsheet.

**Example**

findRSID(15, 79845218)

findRSID(15, 79845218, 79845238, file_path = "output.xlsx")

findRSID(15, 80137560, build= "37")

**Output**

Returns a data table containing variant information including:

- **id:** variant id in rsID format
- **alleles:** variant alleles
- **seq_region_name:** chromosome number
- **start:** starting base pair
- **end:** ending base pair

## findSynonyms()

This function queries the Ensembl REST API for a set of variant identifiers and retrieves any alternative rsIDs reported for each variant. Input variants must be provided as a character vector of valid rsID strings, such as `"rs12345"`. The function returns a `data.table` with one row per input–synonym pair, linking each queried rsID to its corresponding synonym rsID(s), if any are found in Ensembl.

**Function parameters**

- **rsids,** Character, A character vector of variant identifiers in rsID format..
- **build,** Numeric. Genome build to query. Supported values are `37` and `38`. Defaults to `38`.
- **keepRSOnly,** Logical, If TRUE, only synonyms starting with 'rs' will be retained in the output.

**Example**

findSynonyms(c('rs699','rs1234'))

**Output**

Returns a data table containing variant information including:

- **input_rsid:** input variant id in rsID format
- **updated_rsid:** returned variant id from Ensembl
- **synonym:** synonym variant id

## getConfigFile()

Copy a sample configuration file to specified folder.

**Function parameters**

- **dir.path,** The existing folder for copying the file.

**Example**

getConfigFile('/home/user/')

**Output**

A configuration file is saved in the selected folder

## run_annotation()

Run the annotation pipeline on a list of variants.

**Function parameters**

- **configurationFilePath,** The path to the configuration file.
- **verbose,** Whether to display messages in the console. Default: TRUE.

**Example**

output = run_annotation('/home/user/config.ini')

**Output**

A data table containing all variant information is returned based on the user's selected specifications and parameters. Report files in various formats, including text, HTML, Excel, and image, are saved in the output folder.

merge_annotations()

This function loads multiple result objects produced by SNPannotator package. It merges the relevant tables (e.g., input variants, trait association, ...) across all objects and exports the combined results to a single Excel file. Optionally, it can trigger annotation and enrichment analyses using the STRING database.

**Function parameters**

- **file_names,** A list containing Paths to the annotation output **.rds** files to merge.
- **project_name,** The project name used for naming the output Excel file and the log file.
- **STRING,** If **TRUE**, performs annotation and enrichment analysis using STRING for the merged data.
- **…,** Additional arguments passed to downstream functions for extended customization.
  - **network_image,** Default: TRUE
  - **string_server**, Server address to access STRING-DB. Default: <https://string-db.org/api>
  - **graph_layout**, Layout for generating graphs. Default: layout_nicely
  - **required_score,** Threshold of significance to include an interaction, a number between 0 and 1000. Default: 700.
  - **limit,** Limits the number of interaction partners retrieved per protein, a number between 0 and 100,. Default 0.
  - **enrichment_image.** Default: TRUE
  - **functional_enrichment.** Default: TRUE
  - **webLink** Default: TRUE
  - **description**. Default: TRUE
  - **imagetype.** Available items = 'image','svg','highres_image'. Default: 'image'

**Example**

merge_annotations(c("output1.rds", "output2.rds", "output3.rds"), project_name = 'project')

**Output**

Set of report files, including images, text and excel files containing merged relevant tables (e.g., input variants, trait association, ...) across all objects.

run_stringdb_annotation()

This function accepts a vector of gene identifiers and queries the STRING-DB database to retrieve their protein-protein interaction partners. Subsequently, it conducts a functional enrichment analysis to identify significantly overrepresented biological processes, pathways, or molecular functions associated with the interaction network.

**Function parameters**

- **name,** A character string specifying a unique identifier for this analysis run.
- **gene_list,** A character vector of gene symbols (e.g., HGNC symbols or Ensembl gene IDs).
- **required_score,** Threshold of significance to include an interaction, a number between 0 and 1000. Default: 700.
- **limit,** Limits the number of interaction partners retrieved per protein, a number between 0 and 100,. Default 0.
- **…,** Additional arguments passed to downstream functions for extended customization.
  - **network_image.** Default: TRUE
  - **enrichment_image.** Default: TRUE
  - **functional_enrichment.** Default: TRUE
  - **webLink** Default: TRUE
  - **description**. Default: TRUE
  - **imagetype.** Available items = 'image','svg','highres_image'. Default: 'image'

**Example**

run_stringdb_annotation('BC_project', c('BRCA1', 'BRCA2'), limit = 10)

**Output**

Set of report files, including images, text and excel files containing functional enrichment analysis results.
