# Supplementary material for: SNPannotator: automated functional annotation of genetic variants and linked proxies
Source: Bioinformatics. 2026 Aug 24;42(9):btag603. doi: 10.1093/bioinformatics/btag603 (PMC13533565; doi:10.1093/bioinformatics/btag603)
Supplement: Supplementary file 1 [file btag603_supplementary_data.zip › Comparison_Report_R3.docx]

**SUPPLEMENTAL MATERIAL**

**SNPannotator: Automated Functional Annotation of Genetic Variants and Linked Proxies**

Alireza Ani, Ilja M. Nolte, Zoha Kamali, Harold Snieder, Ahmad Vaez

Table of contents

[1. Introduction	2](#_Toc233579851)

[1.1 ANNOVAR	2](#_Toc233579852)

[1.2 MAGMA	2](#_Toc233579853)

[1.3 FUMA	2](#_Toc233579854)

[1.4 SNPannotator	3](#_Toc233579855)

[1.5 Comparison of FUMA and SNPannotator	5](#_Toc233579856)

[2. Methods	6](#_Toc233579857)

[2.1 FUMA runtime environment and configuration	6](#_Toc233579858)

[2.2 SNPannotator package runtime environment and configuration	9](#_Toc233579859)

[3. Results	10](#_Toc233579860)

[3.1 Reported proxy variants	11](#_Toc233579861)

[3.2 Reported genes	14](#_Toc233579862)

[3.3 Reported traits from GWAScatalog	14](#_Toc233579863)

[3.4 Reported Quantitative Trait Loci (QTL)	16](#_Toc233579864)

[3.5 Reported functional enrichment	25](#_Toc233579865)

[3.5.1 FUMA	25](#_Toc233579866)

[3.5.2 SNPannotator	25](#_Toc233579867)

[3.6 Gene set analysis	29](#_Toc233579868)

[3.7 Comparison of key features	30](#_Toc233579869)

[4. Discussion	32](#_Toc233579870)

[5. References	34](#_Toc233579871)

# 1. Introduction

Several tools exist for post-GWAS variant annotation and functional interpretation. These tools differ in their design, input requirements, outputs, and intended use cases. Below we briefly summarize the most commonly referenced tools for variant annotation and functional analysis. SNPannotator was designed to integrate multiple data sources and analytical steps into a unified workflow. Among existing tools, FUMA most closely resembles this scope and design and has a comparable pipeline structure. Therefore, we used it for comparison, as both tools target similar analytical goals despite differing implementations.

## 1.1 ANNOVAR

ANNOVAR is a script-based command-line tool designed to annotate a list of variants against a collection of locally downloaded databases [1]. While it is widely used, it is not a pipeline in the conventional sense, it does not produce integrated reports, visualizations, or structured summaries. Unlike SNPannotator, each annotation database must be downloaded and stored locally, which requires substantial disk space and ongoing manual maintenance to remain up to date. Outputs consist of separate flat files for each query, with no automated integration across annotation sources. There are no built-in graphical outputs, and interpreting results requires additional downstream processing.

## 1.2 MAGMA

MAGMA serves a fundamentally different purpose and is therefore not directly comparable to SNPannotator. Rather than annotating individual variants, MAGMA performs gene-level and pathway-level analysis using GWAS summary statistics [2]. Its primary input is genome-wide association statistics aggregated across genomic regions, not individual SNP identifiers. It is a specialized statistical tool for gene-set analysis and is not designed for variant-level annotation or functional reporting.

## 1.3 FUMA

FUMA is a web-based platform for post-GWAS functional annotation [3]. It accepts GWAS summary statistics and processes them through two parallel workflows: SNP2GENE, which maps variants to genes based on linkage disequilibrium (LD) and genomic proximity, and GENE2FUNC, which performs functional enrichment analysis on the resulting gene set. FUMA produces a comprehensive set of tables and figures automatically and is widely adopted in the GWAS community.

## 1.4 SNPannotator

SNPannotator is an R package for automated post-GWAS analysis that integrates multi-step variant annotation into a single, streamlined pipeline. Starting from a list of lead variants, the pipeline identifies proxy variants in high LD, then systematically queries a curated set of genomic resources including Ensembl [4], the GTEx Portal [5]/eQTL Catalog [6], and STRING-DB [7], to retrieve genomic position, deleteriousness scores, regulatory and clinical annotations, trait associations, expression and splicing quantitative trait loci (eQTLs and sQTLs), and functional enrichment results. All outputs are compiled into structured, user-friendly reports. The package also includes auxiliary functions for standalone variant lookup and LD exploration. Together, these components provide researchers with a practical and efficient framework for deriving biologically meaningful insights from GWAS findings and for prioritizing candidate variants for downstream functional validation. Its principal advantages are summarized below.

**1. Real-time access to current data**

Annotations are retrieved in real time from established, versioned APIs. Users are guaranteed to receive the most up-to-date data available from each source at the time of analysis, without any local database storage or manual maintenance.

**2. Versioned & reproducible results**

Because APIs serve versioned data (e.g., Ensembl release 115), results are reproducible when the same API version is specified. Users may also deliberately query archived database versions to compare results across releases or to match a previously published analysis.

**3. Easy installation & configuration**

The package is installed via CRAN or GitHub with minimal dependencies. No external command-line tools are required. A minimal list of parameters are set in a plain-text configuration file with inline comments and examples, making it straightforward to configure, share, and reproduce analyses.

**4. Structured, user-friendly output**

Results are delivered as color-coded Excel workbooks and an HTML summary report, providing an integrated and interpretable overview of annotation results without requiring additional post-processing.

**5. Synonym & update resolution**

The pipeline automatically checks for updated or synonymous rsIDs via Ensembl, ensuring that deprecated or merged variant identifiers are correctly resolved and do not result in missed annotations.

**6. Batched analysis for large variant lists**

For large input sets, the pipeline supports chunked, batched processing. Results from individual batches can be merged using the built-in merge function, enabling scalable analysis without memory or API rate constraints.

**7. Incremental updates to the reports**

New variants can be appended and annotated without repeating the full analysis. This is particularly useful for iterative analyses or when variants are added following initial submission.

**8. Local execution, no data upload**

All analyses run locally on the user’s machine. No data is uploaded to external servers, preserving data privacy and eliminating dependence on server availability or queue times.

**9. Minimal input requirements**

The package requires only a list of rsIDs as input. No multi-column summary statistics, effect sizes, or p-values are needed. This makes it applicable to scenarios where full summary statistics are unavailable.

**10. Auxiliary annotation utilities**

Independent utility functions are available for targeted use cases outside the main pipeline, including genomic region queries, pairwise LD calculation, proxy variant retrieval, synonym variant retrieval, and standalone STRING-DB enrichment analysis on a user-supplied gene list.

**11. Web interface**

In addition to the R package, a live website (<https://omicslab.org/snpannotator>) offers the auxiliary functions as interactive online tools for quick lookups. Compared with the original data provider websites, this interface is faster and easier to use because it requires only the necessary input parameters and retrieves data through their APIs.

**12. Detailed event logging**

A comprehensive log is generated for each analysis run, recording server queries, API responses, synonym resolutions, and any errors or warnings encountered at each stage.

**13. Guided tutorial**

A detailed step-by-step tutorial is provided, covering recommended parameter choices, interpretation of outputs, and best practices for running a complete post-GWAS annotation pipeline.

**14. Open access & open source**

The package is freely available under a standard open-source license from both CRAN and GitHub, ensuring broad accessibility and community transparency.

## 1.5 Comparison of FUMA and SNPannotator

While FUMA and SNPannotator share a common goal, they differ in their practical design and accessibility on the following points:

- FUMA relies on periodically updated static reference datasets hosted on its servers. Therefore, the currency of the data depends on the provider’s update schedule. In contrast, SNPannotator queries live APIs at the time of analysis, providing access to the most up-to-date data available from each source. Because the data are retrieved without intermediate preprocessing or local modification in SNPannotator, their integrity and consistency with the original provider are preserved. Additionally, users can access previous releases by specifying alternative server URLs corresponding to earlier database versions.
- FUMA processes data on remote servers, requiring users to upload data and wait for server-side job completion. SNPannotator runs entirely on the user’s local machine.
- FUMA requires multi-column GWAS summary statistics as input. A recent version (2.1.5, released 2026-06-26) allows user-submitted lead SNPs with either three columns or rsID only. When only rsID is provided, FUMA retrieves chromosome and position from dbSNP v146, which increases processing time. Notably, the current dbSNP version is 157. SNPannotator requires only a list of rsIDs to query Ensembl API.
- SNPannotator provides detailed per-variant event logs, synonym resolution, and formatted Excel and HTML outputs with color coding which are absent in FUMA’s default output structure.

# 2. Methods

For this comparative analysis, we processed 22 previously identified depression-associated SNPs [8] with both FUMA and SNPannotator. The full SNP list is provided in Table 1.

Table 1. Variants used in the tool comparison analysis.

| **No.** | **rsID** | **Chr** | **Pos^*^** | **No.** | **rsID** | **Chr** | **Pos** |
| --- | --- | --- | --- | --- | --- | --- | --- |
| 1 | rs12407717 | 1 | 30426014 | 12 | rs1265099 | 6 | 31105413 |
| 2 | rs12128108 | 1 | 50293421 | 13 | rs389883 | 6 | 31947460 |
| 3 | rs2422320 | 1 | 73293332 | 14 | rs4976976 | 8 | 143311653 |
| 4 | rs2318763 | 1 | 150115974 | 15 | rs2026194 | 9 | 85082899 |
| 5 | rs16836940 | 1 | 150416913 | 16 | rs4765914 | 12 | 2420377 |
| 6 | rs7597593 | 2 | 185533580 | 17 | rs10774037 | 12 | 2420526 |
| 7 | rs2535629 | 3 | 52833219 | 18 | rs2182139 | 14 | 60149233 |
| 8 | rs1966136 | 3 | 61153578 | 19 | rs9323497 | 14 | 67873128 |
| 9 | rs7714851 | 5 | 164475774 | 20 | rs4906335 | 14 | 104021141 |
| 10 | rs911186 | 6 | 27150599 | 21 | rs4843613 | 16 | 87489698 |
| 11 | rs9368649 | 6 | 30938883 | 22 | rs9951150 | 18 | 52821124 |

* Variant coordinates are reported in GRCh37 (hg19).

## 2.1 FUMA runtime environment and configuration

FUMA analysis was performed using the FUMA web server [9]. Version details and job specifications for the SNP2GENE and GENE2FUNC steps were configured using the web interface. Ensembl v110 and GTEx v8 were selected as the highest versions available.

## 2.2 SNPannotator package runtime environment and configuration

The analysis was performed on a personal computer with the following specifications: R version 4.4.0, Windows 10 Enterprise, Intel® Core i5-7400 processor, 8 GB RAM, and SNPannotator version 1.4.7. Ensembl version 115 was automatically utilized as the current reference. Because the sQTL resource and the latest GTEx v10 in our package are available in the GRCh38 genome build via the GTEx portal, this build was used for QTL analyses. Proxy variant annotation was retained on GRCh37 for comparability with FUMA's variant annotation framework.

# 3. Results

Variants were provided to SNPannotator as a plain text file with one rsID per line. For FUMA, the input list was expanded to include chromosome, position, alleles, and dummy p-value (1E-9) columns, as required by the tested version of the SNP2GENE workflow. FUMA’s web interface reported a queue wait of 2 seconds before the job started, with execution completing in 1 minute and 40 seconds. SNPannotator completed the analysis in 10 minutes and 25 seconds using GRCh38, and 12 minutes and 19 seconds using GRCh37. The shorter runtime for GRCh38 was due to the use of the GTEx portal, which supports batch queries.

FUMA results were delivered as two zip archives. The first, containing the main job output from SNP2GENE pipeline, included 18 tab-delimited text files covering annotations, gene names, risk loci, GWAS Catalog entries, lead SNPs, proxy SNPs, MAGMA reports, analysis parameters, and a README file. The second archive, corresponding to the GENE2FUNC module, contained 12 tab-delimited text files including gene IDs, gene information, GTEx expression data and summarization in tabular and matrix formats, analysis parameters, and a README file.

SNPannotator generated 17 files including:

- 4 tab separated text files (annotation report, eQTL, sQTL and enrichment data)
- 9 figures
- 1 HTML report
- 1 formatted Excel report with 11 sheets (annotations, GWAScatalog, QTL data and summarization, gene information, enrichment report)
- 1 log file
- 1 RDS object file

## 3.1 Reported proxy variants

SNPannotator identified 764 proxy variants for the 22 depression-associated SNPs, while FUMA identified 779. Of these, 726 were shared between the two tools. Details of the overlap are reported in Table 2 and variants classification and types reported in FUMA and SNPannotator are reported in Tables 3-5.

Table 2. Overlap of proxy variants identified by FUMA and SNPannotator.

| **SNP** | **Proxy count in**  **FUMA** | **Proxy count in SNPannotator** | **Overlap** |
| --- | --- | --- | --- |
| rs2318763 | 205 | 198 | 198 |
| rs16836940 | 159 | 153 | 148 |
| rs7714851 | 85 | 83 | 76 |
| rs2026194 | 61 | 62 | 59 |
| rs4976976 | 56 | 54 | 53 |
| rs1966136 | 44 | 43 | 43 |
| rs4906335 | 42 | 40 | 40 |
| rs2422320 | 32 | 40 | 32 |
| rs2535629 | 31 | 29 | 28 |
| rs12128108 | 24 | 20 | 18 |
| rs2182139 | 20 | 20 | 19 |
| rs9323497 | 9 | 1 | 1 |
| rs4843613 | 7 | 9 | 7 |
| rs9951150 | 2 | 3 | 2 |
| rs12407717 | 1 | 1 | 1 |
| rs4765914 | 1 | 1 | 1 |
| rs389883 | 0 | 6 | 0 |
| rs10774037 | 0 | 1 | 0 |
| rs1265099 | 0 | 0 | - |
| rs7597593 | 0 | 0 | - |
| rs911186 | 0 | 0 | - |
| rs9368649 | 0 | 0 | - |
| **Total count** | 779 | 764 | 726 |

Table 3. Variant type for lead and proxy variants reported by FUMA.

| **Variant type** | **N** |
| --- | --- |
| intronic | 459 |
| ncRNA_intronic | 181 |
| intergenic | 122 |
| upstream | 10 |
| downstream | 9 |
| ncRNA_exonic | 7 |
| exonic | 6 |
| UTR3 | 5 |
| UTR5 | 2 |

Table 4. Variant classification for lead and proxy variants reported by SNPannotator.

| **Variant class** | **N** |
| --- | --- |
| SNV | 713 |
| indel | 69 |
| insertion | 3 |
| deletion | 2 |
| sequence_alteration | 1 |

Table 5. Variant type for lead and proxy variants reported by SNPannotator.

| **Variant type** | **N** |
| --- | --- |
| intron_variant | 618 |
| intergenic_variant | 75 |
| upstream_gene_variant | 36 |
| downstream_gene_variant | 20 |
| non_coding_transcript_exon_variant | 16 |
| 3_prime_UTR_variant | 6 |
| missense_variant | 6 |
| regulatory_region_variant | 3 |
| 5_prime_UTR_variant | 2 |
| splice_polypyrimidine_tract_variant | 2 |
| synonymous_variant | 2 |
| splice_region_variant | 1 |
| NA | 1 |

Allele frequencies and LD scores were compared across the 726 overlapping variants. Figure 1-A shows the scatterplot of allele frequency (AF) as reported by SNPannotator against minor allele frequency (MAF) as reported by FUMA. The plot reveals a systematic discrepancy, attributable to the fact that SNPannotator reports the alternate allele frequency from Ensembl. After converting AF values to MAF, by taking 1-AF where AF>0.5, the two measures showed perfect agreement (Figure 1-B).

Figure 1. Comparison of allele frequencies across overlapping proxy variants. **(A)** Alternate allele frequency (AF) from Ensembl reported by SNPannotator versus minor allele frequency (MAF) reported by FUMA. **(B)** After converting AF values to MAF [min(AF, 1−AF)], perfect concordance was observed.

LD score comparison across overlapping variants demonstrated very high concordance with a Pearson correlation exceeding 0.995 (Figure 2).

Figure 2. Scatterplot of LD scores for overlapping proxy variants.

## 3.2 Reported genes

Gene assignments reported by FUMA (colon-separated) and SNPannotator (comma-separated) were parsed and compared on a per-SNP basis. Genes unique to each tool and genes identified by both tools are reported in Table 6. Because a single variant can be assigned to multiple genes, we assessed whether the two tools assigned at least one gene in common to the same variant. Using this approach, both tools consistently identify the same primary genomic context for each variant.

Table 6. Overlap in gene assignment between SNPannotator and FUMA.

|  | **Count** | **Gene symbols** |
| --- | --- | --- |
| Reported by both tools | 30 | *AGBL4, APOPT1, ATP6V1D, BAG5, CACNA1C, CTC-340A15.2, ELAVL4, FHIT, ITIH1, ITIH3, KLC1, MRPS21, OTUD7B, PLEK2, PLEKHO1, PRPF3, RNA5SP459, RP11-15B24.5, RP11-179G8.1, RP11-209A2.1, RP11-458I7.1, RP11-894P9.2, RP4-660H19.1, RPRD2, RTN1, TARS2, TSNARE1, VPS45, ZCCHC14, ZNF804A* |
| Reported only by FUMA | 3 | *GPHN, RP11-492I2.1, RP11-73M18.2* |
| Reported only by SNPannotator | 7 | *CTD-2171N6.1, MAP1LC3B, MUC21, NELFE, PSORS1C1, SKIV2L, STK19* |

## 3.3 Reported traits from GWAScatalog

To compare GWAS Catalog annotations between SNPannotator and FUMA, phenotype terms reported by the two tools were first standardized before comparison. Parentheses were removed and trait labels were converted to lower case to minimize mismatches caused only by differences in capitalization, and the resulting terms were then checked against each other for overlap.

Using this approach, SNPannotator reported at least one phenotype for 90 SNPs, whereas FUMA reported at least one phenotype for 81 SNPs. In terms of overall trait diversity, 96 unique phenotype terms were identified in SNPannotator, compared with 66 unique traits in FUMA. These overlapping and tool-specific phenotype terms are summarized in Table 7.

It should be noted that the GWAS Catalog reports two trait fields: DISEASE/TRAIT (the original reported trait) and MAPPED_TRAIT (a standardized ontology term). For example, entries such as Depression, Depression (quantitative trait), Depression (broad), and Depression in smokers are all mapped to the standardized ontology term ‘unipolar depression’. SNPannotator reports the original DISEASE/TRAIT field as returned by the Ensembl API, whereas FUMA appears to use the MAPPED_TRAIT field. Consequently, discrepancies in trait labels between the two tools reflect differences in trait representation rather than underlying data disagreement.

Table 7. Overlap of GWAS Catalog phenotype terms reported.

|  | **Count** | **Terms** |
| --- | --- | --- |
| Reported by both tools | 63 | anorexia nervosa, attention-deficit/hyperactivity disorder, autism spectrum disorder, bipolar disorder, major depression, obsessive-compulsive disorder, schizophrenia, or tourette syndrome pleiotropy; attention deficit hyperactivity disorder or autism spectrum disorder or intelligence pleiotropy; autism spectrum disorder or schizophrenia; autism spectrum disorder, attention deficit-hyperactivity disorder, bipolar disorder, major depressive disorder, and schizophrenia combined; bipolar disorder; bipolar disorder mtag; bipolar disorder or major depressive disorder; blood protein levels; body mass index; brain morphology mostest; broad depression or bipolar disorder; broad depression or major depressive disorder recurrent; broad depression or major depressive disorder self-reported; broad depression or schizophrenia; cognitive ability, years of educational attainment or schizophrenia pleiotropy; cognitive aspects of educational attainment; cognitive performance; core binding factor acute myeloid leukemia; depression; depression broad; depressive symptom measurement or major depressive disorder; depressive symptoms; depressive symptoms mtag; educational attainment; estimated glomerular filtration rate; estimated glomerular filtration rate creatinine; f-dessert liking derived food-liking factor; f-highly palatable foods liking derived food-liking factor; feeling nervous; general cognitive ability; help-seeking from a gp; highest math class taken mtag; insomnia; intelligence; life satisfaction; major depressive disorder; major depressive disorder mtag; neuroticism; neuroticism mtag; obesity in adult survivors of childhood cancer not exposed to cranial radiation; osteoarthritis hip; osteoarthritis of the hip or knee; osteoarthritis with total hip replacement; post-traumatic stress disorder; risk-taking tendency 4-domain principal component model; schizophrenia; schizophrenia mtag; schizophrenia vs autism spectrum disorder ordinary least squares ols; self-reported math ability; self-reported math ability mtag; serum levels of protein c15orf48; serum levels of protein cdhr3; serum levels of protein itih1; serum levels of protein nppb; serum levels of protein rbl2; smoking initiation; smoking initiation ever regular vs never regular mtag; smoking status; subjective well-being mtag; systolic blood pressure; verbal learning; well-being spectrum multivariate analysis; worry |
| Reported only by FUMA | 3 | age of smoking initiation mtag; smoking cessation mtag; waist-hip ratio |
| Reported only by SNPannotator | 33 | a body shape index; arylamine n-acetyltransferase 1 levels; arylamine n-acetyltransferase 1 levels nat1.12632.14.3; diastolic blood pressure; drinks per week; e3 ubiquitin-protein ligase rnf146 levels; eosinophil counts; height; hemoglobin concentration; hip circumference adjusted for bmi; hiv-1 susceptibility; inter-alpha-trypsin inhibitor heavy chain h1 level in chronic kidney disease with hypertension and no diabetes 7955_195; inter-alpha-trypsin inhibitor heavy chain h1 levels; inter-alpha-trypsin inhibitor heavy chain h1 levels itih1.7955.195.3; inter-alpha-trypsin inhibitor heavy chain h3 levels; janus kinase and microtubule-interacting protein 3 level in chronic kidney disease with hypertension and no diabetes 9068_17; janus kinase and microtubule-interacting protein 3 levels; janus kinase and microtubule-interacting protein 3 levels jakmip3.9068.17.3; mean arterial pressure; nonalcoholic fatty liver disease imputed; normal mucosa of esophagus-specific gene 1 protein levels nmes1.6406.3.3; pulmonary function smoking interaction; retinoblastoma-like protein 2 levels; single ig il-1-related receptor level in chronic kidney disease with hypertension and no diabetes 8326_63; single ig il-1-related receptor levels; single ig il-1-related receptor levels sigirr.8326.63.3; sodium channel subunit beta-2 levels; sun domain-containing protein 3 levels sun3.8852.10.3; surgical hip osteoarthritis; ubiquitin-conjugating enzyme e2 q1 level in chronic kidney disease with hypertension and no diabetes 21732_78; waist-hip index; waist-to-hip ratio adjusted for bmi; zinc finger protein 334 levels |

## 3.4 Reported Quantitative Trait Loci (QTL)

eQTL annotations were available from both FUMA (gtex/v8) and SNPannotator (gtex/v10). The total number of reported eQTL associations and unique genes in each tool are reported in Table 8. We calculated the number of overlapping genes reported by both tools for each lead SNP, providing a direct measure of agreement at gene-level. SNPannotator and FUMA reported 238 and 116 unique genes, respectively, with 99 genes overlapping between the two. It should be noted that SNPannotator enforces a hard limit of 10,000 associations per lead variant to prevent over-representation. This limit was reached for two variants, without which additional associations would have been reported.

Table 8. Number of eQTL associations and unique genes for each lead variant and their overlap.

| **Lead SNP** | **Association count in FUMA** | **Association count in SNPannotator** | **Unique gene count in FUMA** | **Unique gene count in SNPannotator** | **Gene overlap count** |
| --- | --- | --- | --- | --- | --- |
| RS16836940 | 9957 | 10000 | 16 | 18 | 11 |
| RS2318763 | 6058 | 10000 | 17 | 24 | 13 |
| RS4906335 | 4245 | 5887 | 20 | 21 | 17 |
| RS2535629 | 4156 | 5120 | 24 | 31 | 23 |
| RS2182139 | 381 | 445 | 6 | 9 | 6 |
| RS9323497 | 353 | 142 | 8 | 11 | 8 |
| RS4976976 | 130 | 369 | 3 | 5 | 3 |
| RS911186 | 101 | 152 | 21 | 20 | 16 |
| RS1966136 | 65 | 86 | 1 | 1 | 1 |
| RS4843613 | 64 | 135 | 3 | 6 | 3 |
| RS2422320 | 39 | 66 | 2 | 2 | 2 |
| RS12128108 | 18 | 17 | 2 | 2 | 0 |
| RS9951150 | 8 | 8 | 1 | 1 | 1 |
| RS4765914 | 4 | 4 | 2 | 1 | 1 |
| RS10774037 | 0 | 4 | 0 | 1 | 0 |
| RS1265099 | 0 | 207 | 0 | 34 | 0 |
| RS2026194 | 0 | 39 | 0 | 1 | 0 |
| RS389883 | 0 | 4435 | 0 | 73 | 0 |
| RS7714851 | 0 | 69 | 0 | 1 | 0 |
| RS9368649 | 0 | 94 | 0 | 21 | 0 |

eQTL results comparison at the tissue level including the total number of reported associations, the number of unique genes and their overlap is reported in Table 9. The tissue-level bar plots in Figures 3 and 4 visualize this difference.

Table 9. Number of eQTL associations and unique genes identified by FUMA and SNPannotator for each tissue, and the overlap between the two tools.

| **Tissue** | **Association count in FUMA** | **Association count in SNPannotator** | **Unique gene count in FUMA** | **Unique gene count in SNPannotator** | **Gene overlap count** |
| --- | --- | --- | --- | --- | --- |
| cells_cultured_fibroblasts | 2400 | 601 | 41 | 58 | 26 |
| skin_sun_exposed_lower_leg | 1845 | 758 | 31 | 72 | 18 |
| nerve_tibial | 1595 | 2789 | 40 | 93 | 37 |
| artery_tibial | 1592 | 2911 | 36 | 84 | 34 |
| esophagus_muscularis | 1567 | 2381 | 25 | 62 | 24 |
| esophagus_mucosa | 1453 | 500 | 28 | 53 | 15 |
| adipose_subcutaneous | 1223 | 2024 | 35 | 77 | 29 |
| skin_not_sun_exposed_suprapubic | 1041 | 1882 | 25 | 75 | 23 |
| artery_aorta | 954 | 1541 | 30 | 63 | 25 |
| lung | 947 | 426 | 24 | 49 | 16 |
| thyroid | 921 | 2154 | 37 | 87 | 33 |
| brain_cerebellum | 887 | 318 | 14 | 29 | 10 |
| muscle_skeletal | 713 | 1106 | 22 | 60 | 21 |
| testis | 695 | 1258 | 29 | 66 | 26 |
| whole_blood | 684 | 1358 | 23 | 65 | 22 |
| esophagus_gastroesophageal_junction | 628 | 1492 | 21 | 51 | 20 |
| brain_cerebellar_hemisphere | 602 | 243 | 12 | 27 | 8 |
| colon_transverse | 590 | 1023 | 21 | 53 | 18 |
| adipose_visceral_omentum | 545 | 1075 | 20 | 56 | 19 |
| heart_atrial_appendage | 454 | 406 | 17 | 44 | 13 |
| breast_mammary_tissue | 375 | 457 | 20 | 49 | 17 |
| brain_frontal_cortex_ba9 | 348 | 661 | 5 | 25 | 4 |
| heart_left_ventricle | 329 | 603 | 12 | 36 | 12 |
| colon_sigmoid | 307 | 1240 | 13 | 43 | 12 |
| pituitary | 243 | 1038 | 10 | 33 | 10 |
| spleen | 241 | 335 | 11 | 30 | 11 |
| brain_hypothalamus | 217 | 582 | 5 | 26 | 5 |
| brain_cortex | 211 | 245 | 7 | 24 | 6 |
| ovary | 208 | 149 | 7 | 15 | 6 |
| brain_caudate_basal_ganglia | 199 | 674 | 6 | 27 | 5 |
| brain_hippocampus | 190 | 640 | 3 | 21 | 3 |
| pancreas | 180 | 392 | 11 | 33 | 11 |
| cells_ebv-transformed_lymphocytes | 168 | 312 | 5 | 34 | 4 |
| adrenal_gland | 160 | 258 | 10 | 27 | 10 |
| stomach | 151 | 323 | 9 | 28 | 8 |
| artery_coronary | 130 | 174 | 9 | 20 | 7 |
| prostate | 96 | 853 | 7 | 28 | 7 |
| brain_putamen_basal_ganglia | 94 | 167 | 5 | 16 | 3 |
| small_intestine_terminal_ileum | 78 | 102 | 6 | 14 | 6 |
| brain_anterior_cingulate_cortex_ba24 | 69 | 90 | 3 | 10 | 1 |
| brain_nucleus_accumbens_basal_ganglia | 64 | 658 | 5 | 28 | 4 |
| liver | 61 | 175 | 6 | 19 | 5 |
| brain_spinal_cord_cervical_c-1 | 43 | 224 | 3 | 15 | 3 |
| minor_salivary_gland | 29 | 70 | 2 | 6 | 2 |
| brain_amygdala | 28 | 55 | 1 | 7 | 1 |
| vagina | 12 | 98 | 3 | 9 | 2 |
| uterus | 11 | 360 | 1 | 7 | 1 |
| brain_substantia_nigra | 1 | 80 | 1 | 7 | 0 |
| kidney_cortex | 0 | 18 | 0 | 3 | 0 |

Figure 3. eQTL associations count per tissue reported in FUMA.

Figure 4. eQTL associations count per tissue reported in SNPannotator.

The concordance of the reported effect sizes for shared eQTL associations is illustrated in the scatter plot shown in Figure 5, which demonstrates a very strong correlation between the reported effect sizes.

Figure 5. **Scatterplot comparing effect sizes of shared eQTL associations (N=14751)**

sQTL annotations were available only from SNPannotator. Associations count at the lead-SNP and tissue levels of reported in Tables 10 and 11.

Table 10. Number of sQTL associations and unique genes identified by SNPannotator for each lead variant.

| **Lead SNP** | **Association count** | **Unique gene count** |
| --- | --- | --- |
| rs389883 | 8954 | 38 |
| rs4906335 | 8571 | 11 |
| rs2535629 | 7208 | 18 |
| rs4976976 | 3947 | 1 |
| rs16836940 | 1619 | 5 |
| rs2318763 | 1033 | 11 |
| rs1265099 | 343 | 19 |
| rs2182139 | 192 | 6 |
| rs9368649 | 191 | 10 |
| rs1966136 | 129 | 1 |
| rs911186 | 91 | 8 |
| rs7714851 | 68 | 1 |
| rs12128108 | 40 | 2 |
| rs9323497 | 29 | 5 |
| rs2422320 | 22 | 1 |
| rs4843613 | 19 | 1 |

Table 11. Number of sQTL associations and unique genes identified by SNPannotator for each tissue.

| **Tissue** | **Association count** | **Unique gene count** |
| --- | --- | --- |
| artery_tibial | 1533 | 34 |
| testis | 1526 | 57 |
| skin_sun_exposed_lower_leg | 1419 | 42 |
| esophagus_mucosa | 1392 | 25 |
| skin_not_sun_exposed_suprapubic | 1282 | 35 |
| cells_cultured_fibroblasts | 1256 | 26 |
| adipose_subcutaneous | 1185 | 34 |
| thyroid | 1133 | 35 |
| muscle_skeletal | 1075 | 29 |
| nerve_tibial | 1033 | 34 |
| brain_cerebellum | 963 | 22 |
| breast_mammary_tissue | 912 | 25 |
| whole_blood | 842 | 28 |
| lung | 841 | 26 |
| artery_aorta | 817 | 26 |
| heart_atrial_appendage | 751 | 25 |
| cells_ebv-transformed_lymphocytes | 731 | 22 |
| adipose_visceral_omentum | 727 | 25 |
| colon_transverse | 711 | 23 |
| esophagus_muscularis | 662 | 26 |
| colon_sigmoid | 656 | 19 |
| esophagus_gastroesophageal_junction | 651 | 21 |
| adrenal_gland | 646 | 17 |
| pituitary | 632 | 23 |
| artery_coronary | 566 | 16 |
| pancreas | 523 | 14 |
| stomach | 505 | 15 |
| brain_hippocampus | 492 | 14 |
| spleen | 479 | 14 |
| heart_left_ventricle | 476 | 14 |
| brain_cerebellar_hemisphere | 473 | 17 |
| prostate | 465 | 16 |
| brain_putamen_basal_ganglia | 418 | 10 |
| brain_amygdala | 384 | 11 |
| liver | 373 | 13 |
| brain_caudate_basal_ganglia | 365 | 13 |
| brain_spinal_cord_cervical_c-1 | 352 | 9 |
| brain_substantia_nigra | 340 | 8 |
| brain_hypothalamus | 335 | 13 |
| brain_nucleus_accumbens_basal_ganglia | 319 | 16 |
| small_intestine_terminal_ileum | 312 | 9 |
| ovary | 296 | 10 |
| brain_cortex | 271 | 14 |
| minor_salivary_gland | 264 | 7 |
| uterus | 244 | 8 |
| vagina | 235 | 7 |
| brain_frontal_cortex_ba9 | 228 | 13 |
| brain_anterior_cingulate_cortex_ba24 | 223 | 11 |
| bladder | 101 | 4 |
| kidney_cortex | 41 | 1 |

Figure 6. sQTL associations count per tissue reported in SNPannotator.

## 3.5 Reported functional enrichment

### 3.5.1 FUMA

According to the FUMA report files 142 genes were used for enrichment analysis. The report table including category and count in each category are presented in Table 12.

Table 12. Number of statistically significant enriched terms across annotation categories in the FUMA enrichment analysis.

| **Category** | **Count** |
| --- | --- |
| Reactome | 48 |
| GWAScatalog | 38 |
| Canonical_Pathways | 31 |
| Curated_gene_sets | 17 |
| Positional_gene_sets | 6 |
| GO_cc | 2 |

### 3.5.2 SNPannotator

SNPannotator’s gene set (N = 37) was first submitted to STRING-DB for protein interaction network analysis. Applying a minimum interaction score of 0.7 and allowing up to 10 interaction partners per node expanded the gene set to 213 genes. The resulting network, generated by STRING-DB, is shown in Figure 7. Functional enrichment analysis was then performed on this expanded set. The number of enriched terms per biological category is summarized in Table 13. Category-specific enrichment plots, generated by STRING-DB, for Molecular Function, Biological Process, Cellular Component, Diseases, and Tissues are presented in Figures 8–12.

Figure 7. Network visualization of the expanded gene set from STRING-DB.

Table 13. Number of statistically significant enriched terms per functional category returned by STRING-DB enrichment analysis of the mapped gene set in SNPannotator.

| **Category** | **Count** |
| --- | --- |
| Process | 261 |
| Component | 147 |
| COMPARTMENTS | 127 |
| RCTM | 102 |
| PMID | 100 |
| KEGG | 69 |
| NetworkNeighborAL | 65 |
| Function | 61 |
| TISSUES | 46 |
| InterPro | 43 |
| Keyword | 37 |
| WikiPathways | 20 |
| Pfam | 16 |
| DISEASES | 6 |
| SMART | 6 |
| HPO | 6 |

Figure 8. Top enriched Gene Ontology Cellular Component terms for the mapped gene set from STRING-DB ordered by False Discovery Rate (FDR) corrected p-values as part of SNPannotator’s pipeline.

 Figure 9. Top enriched Disease-gene association terms for the mapped gene set from STRING-DB ordered by False Discovery Rate (FDR) corrected p-values as part of SNPannotator’s pipeline.

Figure 10. Top enriched Gene Ontology Molecular Function terms for the mapped gene set from STRING-DB ordered by False Discovery Rate (FDR) corrected p-values as part of SNPannotator’s pipeline.

Figure 11. Top enriched Gene Ontology Biological Process terms for the mapped gene set from STRING-DB ordered by False Discovery Rate (FDR) corrected pvalues as part of SNPannotator’s pipeline.

Figure 12. Top enriched Tissue Expression terms for the mapped gene set from STRING-DB ordered by False Discovery Rate (FDR) corrected p-values as part of SNPannotator’s pipeline.

## 3.6 Gene set analysis

Gene set analysis was performed using the GENE2FUNC module in FUMA. This module uses the mapped gene list as input and performs functional characterization by testing enrichment across biological pathways, tissue expression profiles, and curated gene sets. Enrichment was evaluated against established resources, including MSigDB, WikiPathways, and GTEx-derived differentially expressed gene sets (DEGs) across multiple tissues. The module produced several tab-delimited output files, including the gene list with basic genomic annotation per gene; gene set enrichment results containing pathway names, p-values, adjusted p-values, and overlapping genes for each tested set; differentially expressed gene set results per tissue; tissue-level expression values derived from GTEx v8; and MAGMA-based tissue expression and gene set analysis results.

## 3.7 Comparison of key features

A comparison of key features of FUMA and SNPannotator as well as ANNOVAR is presented in Table 14. Regarding execution speed, the tools differ based on their underlying architecture. FUMA operates on a remote web server, making its speed contingent on server load and network latency, though this is generally fast under normal conditions. SNPannotator relies on real-time API queries to external databases, therefore execution speed is moderate and depends on internet connection stability and the response times of queried servers.

**Proxy variant identification and LD structure.** Both FUMA and SNPannotator returned largely concordant proxy variant sets. Minor differences in reported proxies are attributable to the difference in Ensembl release versions. SNPannotator queried Ensembl release 115, whereas FUMA relied on release 110. Because LD estimates are derived from reference panel data indexed to a specific genome annotation release, version differences can affect LD block boundaries and proxy selection. Despite this, allele frequencies for overlapping variants were identical across both tools, and the observed differences in LD correlation coefficients for shared variants were very small and consistent with a version-driven rather than methodological problem.

**Gene mapping.** Both FUMA and SNPannotator consistently identified the same primary genomic context for each variant, indicating that core gene mapping logic is equivalent between the two pipelines.

**GWAS Catalog annotation.** SNPannotator returned a larger number of associated traits, reflecting its use of a more recent GWAS Catalog build (through Ensembl). Minor discrepancies in trait labels were observed and could be explained by the difference in variable reporting, that is SNPannotator reports the original DISEASE/TRAIT field as returned by Ensembl, while FUMA returned the MAPPED_TRAIT field, which is a standardized ontology term.

**eQTL and sQTL data.** SNPannotator returned more eQTL associations than FUMA, because of using GTEx v10 compared to GTEx v8. For associations reported by both tools, effect size estimates showed strong concordance, with residual differences consistent with the version gap in the reference datasets. sQTL data, derived across multiple tissues, was only available in SNPannotator.

**Functional enrichment analysis.** Enrichment analysis was more comprehensive in SNPannotator (through STRING-DB), covering 16 functional categories, with gene sets, raw p-values, and FDR-adjusted p-values reported for each category. Category-specific enrichment plots and network visualizations were generated as part of the standard output. FUMA performs gene-set analysis through its GENE2FUNC module using MAGMA, which is a robust method but operates on a different statistical framework and uses a distinct set of annotation categories. Therefore, the two approaches are not directly comparable.

**Output structure and reporting.** FUMA delivers results through a web-based interface. Tabular data are exported as a zip archive containing separate tab-delimited files for SNPs, genes, GWAS Catalog entries, and other annotation layers. These files must be manually merged for downstream analyses. Figures are displayed interactively on the web interface and must be saved individually. In contrast, SNPannotator produces a formatted, multi-sheet Excel workbook in which each sheet is self-contained and ready for use without further processing. In addition, an HTML report provides an integrated, user-friendly overview of the most important analytical outputs, including regional association plots, network graphs, etc. The HTML report is suitable for sharing and is designed to be publication-ready.

Table 14. Comparison of key features across commonly used variant annotation tools.

| **Feature** | **FUMA** | **SNPannotator** | **ANNOVAR** |
| --- | --- | --- | --- |
| Integrated end-to-end pipeline | yes | yes | no |
| Execution environment | remote server | local | local |
| Operating system dependency | N/A* | cross-platform | cross-platform |
| Programming requirement | N/A | R | Perl |
| Local storage required | N/A | none | large |
| Data currency | periodically updated | real-time | manual update required |
| Formatted Excel output | no | yes, color-formatted sheets | no |
| HTML report | web-based interface | yes (saved locally) | no |
| Figures | yes (web-based) | yes (png, saved locally) | no |
| Batched analysis | no | yes | limited |
| Execution Speed | Fast; depends on server load and network latency | Moderate; API-based queries depend on internet connection stability and response times | Generally fast; local computation depends on system resources and dataset size |
| Data privacy | data uploaded to remote server | full (local only) | full (local only) |

* N/A, not applicable (web-based)

# 4. Discussion

Post-GWAS functional annotation is an essential step in translating statistical associations into biological insights. Several tools are available for this purpose, with differing designs, input requirements, output formats, and intended use cases. The widely-used FUMA platform is most similar in aim to SNPannotator. Therefore, we compared SNPannotator to FUMA using a list of 22 previously reported depression-associated SNPs. Our comparison suggests that SNPannotator and FUMA perform equivalently for proxy variant identification and gene mapping, with observed discrepancies attributable to differences in database release versions rather than methodological divergence. For GWAS Catalog annotation, eQTL data, and sQTL analysis, SNPannotator yields richer results because it uses more recent data sources in real time through live API calls. Functional enrichment analysis is broader in scope within SNPannotator, while gene-set analysis via MAGMA was currently only available through FUMA.

Although SNPannotator is comparatively slower than FUMA, the additional runtime is unlikely to pose a meaningful bottleneck in practice, as post-GWAS annotation is typically performed only once per study. From a usability perspective, SNPannotator operates entirely within the R environment, requires only a configuration file and a minimal input. All annotation layers are integrated into a single R object and exported as a structured, multi-tab Excel workbook and HTML file. Beyond core annotation, it provides additional utility functions for variant-level operations, such as synonym resolution and proxy retrieval based on LD thresholds. It also supports batched and incremental analysis. An interactive network visualization feature is also currently under development (preview available at https://omicslab.org/snpannotator), allowing users to explore networks dynamically. Taken together, SNPannotator has certain advantages compared to FUMA for post-GWAS variant annotation and interpretation, making it a practical option for researchers seeking to extract biological insights from GWAS results.

# 5. References

[1]	Wang K, Li M, Hakonarson H. ANNOVAR: functional annotation of genetic variants from high-throughput sequencing data. *Nucleic Acids Research* 2010; 38: e164.

[2]	de Leeuw CA, Mooij JM, Heskes T, et al. MAGMA: Generalized Gene-Set Analysis of GWAS Data. *PLoS Comput Biol* 2015; 11: e1004219.

[3]	Watanabe K, Taskesen E, van Bochoven A, et al. Functional mapping and annotation of genetic associations with FUMA. *Nat Commun* 2017; 8: 1826.

[4]	Dyer SC, Austine-Orimoloye O, Azov AG, et al. Ensembl 2025. *Nucleic Acids Research* 2025; 53: D948–D957.

[5]	Lonsdale J, Thomas J, Salvatore M, et al. The Genotype-Tissue Expression (GTEx) project. *Nat Genet* 2013; 45: 580–585.

[6]	Kerimov N, Tambets R, Hayhurst JD, et al. eQTL Catalogue 2023: New datasets, X chromosome QTLs, and improved detection and visualisation of transcript-level QTLs. *PLOS Genetics* 2023; 19: e1010932.

[7]	Szklarczyk D, Kirsch R, Koutrouli M, et al. The STRING database in 2023: protein–protein association networks and functional enrichment analyses for any sequenced genome of interest. *Nucleic Acids Research* 2023; 51: D638–D646.

[8]	Amare AT, Vaez A, Hsu Y-H, et al. Bivariate genome-wide association analyses of the broad depression phenotype combined with major depressive disorder, bipolar disorder or schizophrenia reveal eight novel genetic loci for depression. *Mol Psychiatry* 2020; 25: 1420–1429.

[9]	Watanabe K. General overview of FUMAGWAS. *Functional Mapping and Annotation of Genome-wide association studies*, https://fuma.ctglab.nl (2025, accessed 27 September 2025).
