# Supplementary material for: SNPannotator: automated functional annotation of genetic variants and linked proxies
Source: Bioinformatics. 2026 Aug 24;42(9):btag603. doi: 10.1093/bioinformatics/btag603 (PMC13533565; doi:10.1093/bioinformatics/btag603)
Supplement: Supplementary file 1 [file btag603_supplementary_data.zip › demo.html]

InSilico Sequencing Report


# InSilico Sequencing Report

### SNPannotator package

---

#### Parameters

**Population:** 1000GENOMES:phase\_3:EUR

**Genomic build:** GRCH37

**R2:** 0.8

**Window-size:** 500 Kb

---

#### Variants information

1. rs2422320
2. rs7714851
3. rs9368649
4. rs9323497
5. rs16836940
6. rs2535629
7. rs4765914
8. rs12407717
9. rs12128108
10. rs2318763
11. rs7597593
12. rs1966136
13. rs911186
14. rs1265099
15. rs389883
16. rs4976976
17. rs2026194
18. rs10774037
19. rs2182139
20. rs4906335
21. rs4843613
22. rs9951150

#### Figures

- Variant-trait association
  network
- eQTL association network
- Interaction network
- Functional enrichment
  visualizations

---

Variant ID: rs2422320  
Position: 
1:73293332 (1p31.1)  
Most severe consequence:  Intron Variant  
Proxy
Variants:  40   
  
Mapped genes

RP4-660H19.1

  
Associated phenotypes

Autism spectrum disorder or schizophrenia

Broad depression or major depressive disorder self-reported

Insomnia

Major depressive disorder

Schizophrenia

Schizophrenia mtag

Schizophrenia vs autism spectrum disorder ordinary least squares ols

  

Variant types

| Most severe consequence | count |
| --- | --- |
| intron\_variant | 41 |

  
  

Most deleterious variants

| rsID | CADD score | LD | Gene |
| --- | --- | --- | --- |
| rs12137869 | 11.69 | 1 | RP4-660H19.1 |
| rs1949888 | 9.484 | 0.883908 | RP4-660H19.1 |
| rs11210070 | 9.129 | 0.992049 | RP4-660H19.1 |
| rs12044362 | 8.454 | 0.819555 | RP4-660H19.1 |
| rs10889993 | 8.19 | 0.819555 | RP4-660H19.1 |
| rs12117216 | 5.627 | 0.992049 | RP4-660H19.1 |

  
  
  


---

Variant ID: rs7714851  
Position: 
5:164475774 (5q34)  
Most severe consequence:  Intron Variant  
Proxy
Variants:  83   
  
Mapped genes

CTC-340A15.2

  
Associated phenotypes

Bipolar disorder or major depressive disorder

Broad depression or major depressive disorder self-reported

Depression

Depression (broad)

Depressive symptoms

Depressive symptoms (mtag)

Help-seeking from a gp

Life satisfaction

Major depressive disorder

Major depressive disorder mtag

Neuroticism

Neuroticism (mtag)

Post-traumatic stress disorder

Subjective well-being (mtag)

Well-being spectrum multivariate analysis

  

Variant types

| Most severe consequence | count |
| --- | --- |
| intron\_variant | 84 |

  
  

Most deleterious variants

| rsID | CADD score | LD | Gene |
| --- | --- | --- | --- |
| rs4543289 | 15.26 | 0.937939 | CTC-340A15.2 |
| rs4552634 | 15.03 | 0.937939 | CTC-340A15.2 |
| rs10035449 | 11.43 | 0.937939 | CTC-340A15.2 |
| rs7721129 | 10.78 | 0.937939 | CTC-340A15.2 |
| rs7443123 | 10.41 | 0.988139 | CTC-340A15.2 |
| rs7442891 | 6.472 | 0.988139 | CTC-340A15.2 |

  
  
  


---

Variant ID: rs9368649  
Position: 
6:30938883 (6p21.33)  
Most severe consequence:  Intergenic Variant  
Proxy
Variants:  0   
  
Mapped genes

MUC21

  
Associated phenotypes

Broad depression or major depressive disorder self-reported

Pulmonary function (smoking interaction)

  

Variant types

| Most severe consequence | count |
| --- | --- |
| intergenic\_variant | 1 |

  
  

Most deleterious variants

| rsID | CADD score | LD | Gene |
| --- | --- | --- | --- |
| rs9368649 | 8.424 | 1 | MUC21 |

  
  
  


---

Variant ID: rs9323497  
Position: 
14:67873128 (14q23.3)  
Most severe consequence:  Intron Variant  
Proxy
Variants:  1   
  
Mapped genes

ATP6V1D, PLEK2

  
Associated phenotypes

Broad depression or major depressive disorder recurrent

Depressive symptom measurement or major depressive disorder

  

Variant types

| Most severe consequence | count |
| --- | --- |
| intron\_variant | 2 |

  
  

Most deleterious variants

| rsID | CADD score | LD | Gene |
| --- | --- | --- | --- |
| rs57154550 | 0.963 | 0.943091 | ATP6V1D |
| rs9323497 | 0.152 | 1 | PLEK2 |

  
  
  


---

Variant ID: rs16836940  
Position:
 1:150416913 (1q21.3)  
Most severe consequence:  Intron Variant  
Proxy
Variants:  153   
  
Mapped genes

MRPS21, PRPF3, RPRD2, TARS2

  
Associated phenotypes

Broad depression or bipolar disorder

Drinks per week

Height

  

Variant types

| Most severe consequence | count |
| --- | --- |
| intron\_variant | 130 |
| downstream\_gene\_variant | 5 |
| intergenic\_variant | 2 |
| upstream\_gene\_variant | 10 |
| 3\_prime\_UTR\_variant | 4 |
| non\_coding\_transcript\_exon\_variant | 2 |
| 5\_prime\_UTR\_variant | 1 |

  
  

Most deleterious variants

| rsID | CADD score | LD | Gene |
| --- | --- | --- | --- |
| rs36023386 | 13.31 | 0.981981 | PRPF3 |
| rs41264059 | 13.09 | 0.975988 | TARS2 |
| rs41264139 | 12.18 | 0.975988 | TARS2 |
| rs12121966 | 11.69 | 0.981981 | RPRD2 |
| rs16836143 | 11.06 | 0.975988 | PRPF3 |
| rs66513313 | 11 | 0.987994 | RPRD2 |

  
  
  


---

Variant ID: rs2535629  
Position: 
3:52833219 (3p21.1)  
Most severe consequence:  Intron Variant  
Proxy
Variants:  30   
  
Mapped genes

ITIH1, ITIH3

  
Associated phenotypes

|  |  |  |
| --- | --- | --- |
| Arylamine n-acetyltransferase 1 levels | Janus kinase and microtubule-interacting protein 3 level in chronic kidney disease with hypertension and no diabetes 9068\_17 | Serum levels of protein rbl2 |
| Arylamine n-acetyltransferase 1 levels nat1.12632.14.3 | Janus kinase and microtubule-interacting protein 3 levels | Single ig il-1-related receptor level in chronic kidney disease with hypertension and no diabetes 8326\_63 |
| Autism spectrum disorder, attention deficit-hyperactivity disorder, bipolar disorder, major depressive disorder, and schizophrenia (combined) | Janus kinase and microtubule-interacting protein 3 levels jakmip3.9068.17.3 | Single ig il-1-related receptor levels |
| Bipolar disorder | Normal mucosa of esophagus-specific gene 1 protein levels nmes1.6406.3.3 | Single ig il-1-related receptor levels sigirr.8326.63.3 |
| Blood protein levels | Osteoarthritis (hip) | Smoking initiation |
| Body mass index | Osteoarthritis (with total hip replacement) | Sodium channel subunit beta-2 levels |
| Broad depression or bipolar disorder | Osteoarthritis of the hip or knee | Sun domain-containing protein 3 levels sun3.8852.10.3 |
| Broad depression or schizophrenia | Retinoblastoma-like protein 2 levels | Surgical hip osteoarthritis |
| E3 ubiquitin-protein ligase rnf146 levels | Risk-taking tendency 4-domain principal component model | Ubiquitin-conjugating enzyme e2 q1 level in chronic kidney disease with hypertension and no diabetes 21732\_78 |
| Height | Schizophrenia | Verbal learning |
| Inter-alpha-trypsin inhibitor heavy chain h1 level in chronic kidney disease with hypertension and no diabetes 7955\_195 | Serum levels of protein c15orf48 | Worry |
| Inter-alpha-trypsin inhibitor heavy chain h1 levels | Serum levels of protein cdhr3 | Zinc finger protein 334 levels |
| Inter-alpha-trypsin inhibitor heavy chain h1 levels itih1.7955.195.3 | Serum levels of protein itih1 |  |
| Inter-alpha-trypsin inhibitor heavy chain h3 levels | Serum levels of protein nppb |  |

  

Variant types

| Most severe consequence | count |
| --- | --- |
| intron\_variant | 12 |
| upstream\_gene\_variant | 10 |
| splice\_polypyrimidine\_tract\_variant | 1 |
| non\_coding\_transcript\_exon\_variant | 3 |
| splice\_region\_variant | 1 |
| synonymous\_variant | 1 |
| missense\_variant | 3 |

  

Missense variants

| rsID | CADD score | LD | Gene |
| --- | --- | --- | --- |
| rs678 | 26.4 | 0.842288 | ITIH1 |
| rs678 | 26.4 | 0.842288 | ITIH1 |
| rs1042779 | 13.49 | 0.840022 | ITIH1 |

  

Most deleterious variants

| rsID | CADD score | LD | Gene |
| --- | --- | --- | --- |
| rs678 | 26.4 | 0.842288 | ITIH1 |
| rs678 | 26.4 | 0.842288 | ITIH1 |
| rs2286798 | 15.53 | 0.839302 | ITIH1 |
| rs1042779 | 13.49 | 0.840022 | ITIH1 |
| rs1075653 | 10.78 | 0.846029 | ITIH1 |
| rs4687552 | 9.8 | 0.886509 | ITIH3 |

  
  
  


---

Variant ID: rs4765914  
Position: 
12:2420377 (12p13.33)  
Most severe consequence:  Intron Variant  
Proxy
Variants:  1   
  
Mapped genes

CACNA1C

  
Associated phenotypes

Broad depression or bipolar disorder

Broad depression or schizophrenia

  

Variant types

| Most severe consequence | count |
| --- | --- |
| intron\_variant | 2 |

  
  

Most deleterious variants

| rsID | CADD score | LD | Gene |
| --- | --- | --- | --- |
| rs4765914 | 12.75 | 1 | CACNA1C |
| rs10774037 | 2.391 | 0.988239 | CACNA1C |

  
  
  


---

Variant ID: rs12407717  
Position:
 1:30426014 (1p35.2)  
Most severe consequence:  Intergenic Variant  
Proxy
Variants:  1   
  
Mapped genes

RP11-179G8.1

  
Associated phenotypes

Broad depression or schizophrenia

Core binding factor acute myeloid leukemia

  

Variant types

| Most severe consequence | count |
| --- | --- |
| intergenic\_variant | 2 |

  
  

Most deleterious variants

| rsID | CADD score | LD | Gene |
| --- | --- | --- | --- |
| rs12407717 | 2.006 | 1 | RP11-179G8.1 |
| rs4949232 | 0.302 | 0.964674 | RP11-179G8.1 |

  
  
  


---

Variant ID: rs12128108  
Position:
 1:50293421 (1p33)  
Most severe consequence:  Intron Variant  
Proxy
Variants:  20   
  
Mapped genes

AGBL4, ELAVL4

  
Associated phenotypes

Body mass index

Broad depression or schizophrenia

Insomnia

Schizophrenia

Smoking initiation

Smoking initiation ever regular vs never regular mtag

Smoking status

  

Variant types

| Most severe consequence | count |
| --- | --- |
| intron\_variant | 21 |

  
  

Most deleterious variants

| rsID | CADD score | LD | Gene |
| --- | --- | --- | --- |
| rs12118183 | 17.25 | 0.955506 | ELAVL4 |
| rs56184305 | 11.35 | 0.993571 | AGBL4 |
| rs11205679 | 7.481 | 0.962 | ELAVL4 |
| rs11205658 | 7.408 | 0.993571 | AGBL4 |
| rs12137651 | 5.794 | 0.993571 | AGBL4 |
| rs11205659 | 4.751 | 0.993571 | AGBL4 |

  
  
  


---

Variant ID: rs2318763  
Position: 
1:150115974 (1q21.2)  
Most severe consequence:  Intron Variant  
Proxy
Variants:  198   
  
Mapped genes

OTUD7B, PLEKHO1, RP11-458I7.1, VPS45

  
Associated phenotypes

Anorexia nervosa, attention-deficit/hyperactivity disorder, autism
spectrum disorder, bipolar disorder, major depression,
obsessive-compulsive disorder, schizophrenia, or tourette syndrome
pleiotropy

Bipolar disorder

Bipolar disorder mtag

Brain morphology mostest

Broad depression or schizophrenia

Cognitive ability, years of educational attainment or schizophrenia
pleiotropy

Estimated glomerular filtration rate

Estimated glomerular filtration rate creatinine

Highest math class taken mtag

Obesity in adult survivors of childhood cancer not exposed to cranial
radiation

Schizophrenia

Schizophrenia mtag

Self-reported math ability

Self-reported math ability mtag

  

Variant types

| Most severe consequence | count |
| --- | --- |
| intron\_variant | 92 |
| upstream\_gene\_variant | 15 |
| intergenic\_variant | 66 |
| non\_coding\_transcript\_exon\_variant | 7 |
| downstream\_gene\_variant | 14 |
| 5\_prime\_UTR\_variant | 1 |
| ? | 1 |
| regulatory\_region\_variant | 3 |

  
  

Most deleterious variants

| rsID | CADD score | LD | Gene |
| --- | --- | --- | --- |
| rs2039800 | 17.63 | 0.95916 | VPS45 |
| rs72694923 | 15.71 | 0.985943 | VPS45 |
| rs72694945 | 15.27 | 0.985943 | VPS45 |
| rs12063329 | 14.51 | 0.97891 | OTUD7B |
| rs6666959 | 12.32 | 0.95916 | VPS45 |
| rs72694928 | 11.67 | 0.985943 | VPS45 |

  
  
  


---

Variant ID: rs7597593  
Position: 
2:185533580 (2q32.1)  
Most severe consequence:  Intron Variant  
Proxy
Variants:  0   
  
Mapped genes

ZNF804A

  
Associated phenotypes

Autism spectrum disorder, attention deficit-hyperactivity disorder,
bipolar disorder, major depressive disorder, and schizophrenia
(combined)

Broad depression or schizophrenia

Schizophrenia

  

Variant types

| Most severe consequence | count |
| --- | --- |
| intron\_variant | 1 |

  
  

Most deleterious variants

| rsID | CADD score | LD | Gene |
| --- | --- | --- | --- |
| rs7597593 | 2.507 | 1 | ZNF804A |

  
  
  


---

Variant ID: rs1966136  
Position: 
3:61153578 (3p14.2)  
Most severe consequence:  Intron Variant  
Proxy
Variants:  43   
  
Mapped genes

FHIT

  
Associated phenotypes

Body mass index

Broad depression or schizophrenia

Major depressive disorder mtag

  

Variant types

| Most severe consequence | count |
| --- | --- |
| intron\_variant | 44 |

  
  

Most deleterious variants

| rsID | CADD score | LD | Gene |
| --- | --- | --- | --- |
| rs11130820 | 15.62 | 0.827592 | FHIT |
| rs10049287 | 13.26 | 0.995925 | FHIT |
| rs9816087 | 12.78 | 0.827592 | FHIT |
| rs2886377 | 9.445 | 0.849878 | FHIT |
| rs13320646 | 8.202 | 0.963501 | FHIT |
| rs9871451 | 7.563 | 0.987775 | FHIT |

  
  
  


---

Variant ID: rs911186  
Position: 
6:27150599 (6p22.1)  
Most severe consequence:  Intergenic Variant  
Proxy
Variants:  0   
  
Mapped genes

RP11-209A2.1

  
Associated phenotypes

Autism spectrum disorder or schizophrenia

Broad depression or schizophrenia

Hemoglobin concentration

  

Variant types

| Most severe consequence | count |
| --- | --- |
| intergenic\_variant | 1 |

  
  

Most deleterious variants

| rsID | CADD score | LD | Gene |
| --- | --- | --- | --- |
| rs911186 | 10.14 | 1 | RP11-209A2.1 |

  
  
  


---

Variant ID: rs1265099  
Position: 
6:31105413 (6p21.33)  
Most severe consequence:  3
Prime UTR Variant  
Proxy Variants: 
0   
  
Mapped
genes

PSORS1C1

  
Associated phenotypes

Broad depression or schizophrenia

Hiv-1 susceptibility

  

Variant types

| Most severe consequence | count |
| --- | --- |
| 3\_prime\_UTR\_variant | 1 |

  
  

Most deleterious variants

| rsID | CADD score | LD | Gene |
| --- | --- | --- | --- |
| rs1265099 | 6.114 | 1 | PSORS1C1 |

  
  
  


---

Variant ID: rs389883  
Position: 
6:31947460 (6p21.33)  
Most severe consequence:  Non
Coding Transcript Exon Variant  
Proxy
Variants:  7   
  
Mapped genes

NELFE, SKIV2L, STK19

  
Associated phenotypes

A body shape index

Broad depression or schizophrenia

Diastolic blood pressure

Eosinophil counts

Hip circumference adjusted for bmi

Mean arterial pressure

Nonalcoholic fatty liver disease imputed

Waist-hip index

Waist-to-hip ratio adjusted for bmi

  

Variant types

| Most severe consequence | count |
| --- | --- |
| non\_coding\_transcript\_exon\_variant | 2 |
| splice\_polypyrimidine\_tract\_variant | 1 |
| intron\_variant | 3 |
| missense\_variant | 2 |

  

Missense variants

| rsID | CADD score | LD | Gene |
| --- | --- | --- | --- |
| rs437179 | 13.26 | 0.994151 | SKIV2L |
| rs437179 | 13.26 | 0.994151 | SKIV2L |

  

Most deleterious variants

| rsID | CADD score | LD | Gene |
| --- | --- | --- | --- |
| rs437179 | 13.26 | 0.994151 | SKIV2L |
| rs437179 | 13.26 | 0.994151 | SKIV2L |
| rs419788 | 9.316 | 0.994151 | SKIV2L |
| rs386480 | 6.463 | 1 | STK19 |
| rs389883 | 5.207 | 1 | STK19 |
| rs440454 | 3.767 | 0.994151 | SKIV2L |

  
  
  


---

Variant ID: rs4976976  
Position: 
8:143311653 (8q24.3)  
Most severe consequence:  Intron Variant  
Proxy
Variants:  54   
  
Mapped genes

TSNARE1

  
Associated phenotypes

Attention deficit hyperactivity disorder or autism spectrum disorder or
intelligence pleiotropy

Bipolar disorder mtag

Broad depression or schizophrenia

Cognitive aspects of educational attainment

Cognitive performance

F-dessert liking derived food-liking factor

F-highly palatable foods liking derived food-liking factor

General cognitive ability

Intelligence

Schizophrenia vs autism spectrum disorder ordinary least squares ols

Systolic blood pressure

Worry

  

Variant types

| Most severe consequence | count |
| --- | --- |
| intron\_variant | 55 |

  
  

Most deleterious variants

| rsID | CADD score | LD | Gene |
| --- | --- | --- | --- |
| rs8180914 | 10.6 | 0.882751 | TSNARE1 |
| rs6985751 | 6.335 | 0.876196 | TSNARE1 |
| rs11167136 | 5.403 | 0.894975 | TSNARE1 |
| rs4458978 | 4.564 | 0.899282 | TSNARE1 |
| rs34259915 | 4.19 | 0.842764 | TSNARE1 |
| rs4976977 | 4.113 | 0.895648 | TSNARE1 |

  
  
  


---

Variant ID: rs2026194  
Position: 
9:85082899 (9q21.32)  
Most severe consequence:  Intron Variant  
Proxy
Variants:  62   
  
Mapped genes

RP11-15B24.5

  

Variant types

| Most severe consequence | count |
| --- | --- |
| intron\_variant | 62 |
| non\_coding\_transcript\_exon\_variant | 1 |

  
  

Most deleterious variants

| rsID | CADD score | LD | Gene |
| --- | --- | --- | --- |
| rs2026194 | 18.89 | 1 | RP11-15B24.5 |
| rs7048952 | 16.93 | 0.824339 | RP11-15B24.5 |
| rs11139576 | 12.27 | 0.989831 | RP11-15B24.5 |
| rs7467255 | 11.67 | 0.969512 | RP11-15B24.5 |
| rs12555428 | 8.348 | 0.989831 | RP11-15B24.5 |
| rs7857318 | 7.865 | 0.989831 | RP11-15B24.5 |

  
  
  


---

Variant ID: rs10774037  
Position:
 12:2420526 (12p13.33)  
Most severe consequence:  Intron Variant  
Proxy
Variants:  1   
  
Mapped genes

CACNA1C

  
Associated phenotypes

Broad depression or bipolar disorder

Broad depression or schizophrenia

  

Variant types

| Most severe consequence | count |
| --- | --- |
| intron\_variant | 2 |

  
  

Most deleterious variants

| rsID | CADD score | LD | Gene |
| --- | --- | --- | --- |
| rs4765914 | 12.75 | 0.988239 | CACNA1C |
| rs10774037 | 2.391 | 1 | CACNA1C |

  
  
  


---

Variant ID: rs2182139  
Position: 
14:60149233 (14q23.1)  
Most severe consequence:  Intron Variant  
Proxy
Variants:  20   
  
Mapped genes

RTN1

  
Associated phenotypes

Bipolar disorder or major depressive disorder

Broad depression or schizophrenia

Depression

  

Variant types

| Most severe consequence | count |
| --- | --- |
| intron\_variant | 21 |

  
  

Most deleterious variants

| rsID | CADD score | LD | Gene |
| --- | --- | --- | --- |
| rs1952042 | 13.85 | 0.98968 | RTN1 |
| rs1952039 | 12.66 | 1 | RTN1 |
| rs2182140 | 11.81 | 1 | RTN1 |
| rs1956351 | 11.09 | 1 | RTN1 |
| rs1952033 | 10.61 | 0.994833 | RTN1 |
| rs1957985 | 6.37 | 1 | RTN1 |

  
  
  


---

Variant ID: rs4906335  
Position: 
14:104021141 (14q32.33)  
Most severe consequence:  Intron Variant  
Proxy
Variants:  40   
  
Mapped genes

APOPT1, BAG5, KLC1, RP11-894P9.2

  
Associated phenotypes

Autism spectrum disorder or schizophrenia

Bipolar disorder mtag

Broad depression or schizophrenia

Cognitive ability, years of educational attainment or schizophrenia
pleiotropy

Schizophrenia

Schizophrenia mtag

  

Variant types

| Most severe consequence | count |
| --- | --- |
| intron\_variant | 37 |
| 3\_prime\_UTR\_variant | 1 |
| synonymous\_variant | 1 |
| missense\_variant | 1 |
| non\_coding\_transcript\_exon\_variant | 1 |

  

Missense variants

| rsID | CADD score | LD | Gene |
| --- | --- | --- | --- |
| rs2274267 | 7.159 | 0.959522 | KLC1 |

  

Most deleterious variants

| rsID | CADD score | LD | Gene |
| --- | --- | --- | --- |
| rs35498576 | 12.29 | 1 | BAG5 |
| rs11844466 | 7.653 | 0.82089 | KLC1 |
| rs2274267 | 7.159 | 0.959522 | KLC1 |
| rs35496194 | 6.348 | 0.964606 | APOPT1,KLC1 |
| rs55751606 | 5.661 | 0.974739 | BAG5,KLC1 |
| rs11851616 | 5.309 | 0.825059 | KLC1 |

  
  
  


---

Variant ID: rs4843613  
Position: 
16:87489698 (16q24.2)  
Most severe consequence:  Intron Variant  
Proxy
Variants:  9   
  
Mapped genes

MAP1LC3B, ZCCHC14

  
Associated phenotypes

Broad depression or schizophrenia

Feeling nervous

Schizophrenia

Worry

  

Variant types

| Most severe consequence | count |
| --- | --- |
| intron\_variant | 9 |
| downstream\_gene\_variant | 1 |

  
  

Most deleterious variants

| rsID | CADD score | LD | Gene |
| --- | --- | --- | --- |
| rs9923598 | 5.835 | 0.82277 | ZCCHC14 |
| rs7194676 | 2.91 | 0.993571 | ZCCHC14 |
| rs4843611 | 2.485 | 0.812214 | ZCCHC14 |
| rs1816113 | 1.81 | 0.816674 | MAP1LC3B,ZCCHC14 |
| rs7200522 | 0.918 | 0.89943 | ZCCHC14 |
| rs8050237 | 0.646 | 0.938589 | ZCCHC14 |

  
  
  


---

Variant ID: rs9951150  
Position: 
18:52821124 (18q21.2)  
Most severe consequence:  Intergenic Variant  
Proxy
Variants:  3   
  
Mapped genes

CTD-2171N6.1, RNA5SP459

  
Associated phenotypes

Autism spectrum disorder, attention deficit-hyperactivity disorder,
bipolar disorder, major depressive disorder, and schizophrenia
(combined)

Broad depression or schizophrenia

Educational attainment

Neuroticism

  

Variant types

| Most severe consequence | count |
| --- | --- |
| intergenic\_variant | 3 |
| upstream\_gene\_variant | 1 |

  
  

Most deleterious variants

| rsID | CADD score | LD | Gene |
| --- | --- | --- | --- |
| rs1011339 | 0.787 | 0.996024 | RNA5SP459 |
| rs9951150 | 0.068 | 1 | RNA5SP459 |
| rs11433836 |  | 1 | CTD-2171N6.1 |
| rs35486984 |  | 0.94858 | RNA5SP459 |

  
  
  


---

### Variant-trait association network

Right-click to open full-size image in new tab or save.

  

---

### eQTL association network

Right-click to open full-size image in new tab or save.

  

---

### sQTL association network

Right-click to open full-size image in new tab or save.

  

---

### STRING DB report

#### Interaction network

Right-click to open full-size image in new tab or save.

  

---

#### Functional enrichment visualizations

  

---


  

---


  

---


  

---


  

---

Link
to STRING website


---

Generated by **SNPannotator** [v.1.4.7] (05:12:50 PM - Jun 04
2026)

↑
